# Host-linked virome assembly and turnover predict bacterial community structure in wastewater treatment systems

**DOI:** 10.1093/ismejo/wrag120

**Published:** 2026-05-12

**Authors:** Jinjin Yu, Siang Nee Tang, Patrick K H Lee

**Affiliations:** School of Energy and Environment, City University of Hong Kong, Hong Kong SAR, China; Facility Management and Environmental Engineering, TAL Group, Hong Kong SAR, China; School of Energy and Environment and State Key Laboratory of Marine Environmental Health, City University of Hong Kong, Hong Kong SAR, China; Climate Impact and Environmental Resilience Research Center, City University of Hong Kong, Hong Kong SAR, China

**Keywords:** viral ecology, virus–host interactions, genetic microdiversity, viral community assembly, predictive modeling, engineered wastewater ecosystems

## Abstract

Viruses play crucial roles in bacterial ecology and evolution through virus–host interactions; however, their distribution, assembly mechanisms, and temporal turnover remain underexplored in engineered ecosystems. In the present study, we used activated sludge (AS) and anaerobic treatment (AT) reactors from four full-scale industrial textile wastewater treatment plants as model ecosystems, integrating metagenomics, macroecological modeling, and deep learning to characterize viral structure, dynamics, and host interactions. A total of 1046 and 1386 high-quality viral operational taxonomic units were recovered from AS and AT systems, respectively, and most were affiliated with *Caudoviricetes*. Viral composition and genetic microdiversity were highly plant-specific and shaped by environmental selection and host interactions. Lognormal species abundance distributions and deviations from neutral expectations indicated deterministic assembly. Virulent viruses exhibited faster temporal turnover than temperate viruses. Viral co-occurrence networks showed strong plant-specific modularity and greater temporal stability than bacterial networks, suggesting that they play a stabilizing role in community dynamics. Tight virus–host abundance coupling and gene-level signatures of host-linked selection indicated ongoing coevolutionary interactions. A deep learning model accurately predicted bacterial community dynamics from viral composition at both the taxon and sample levels, highlighting the ecological relevance of viral signatures. Together, these findings reveal dynamic, plant-specific viromes tightly coupled to bacterial communities and highlight viral signatures as potential indicators for monitoring engineered ecosystems. Incorporating viral ecology into microbial management could enhance the stability, resilience, and functional performance of engineered ecosystems.

## Introduction

Biological wastewater treatment plants (WWTPs) are widely implemented engineered ecosystems relying on diverse microbial consortia, including bacteria, fungi, protozoa, and viruses, to transform organic matter, nutrients, and micropollutants [1, 2]. Although bacteria are primary drivers of these processes [3], viruses, particularly bacteriophages, have received limited attention and are viewed as passive components [4, 5]. Evidence indicates that viruses shape bacterial communities through lytic infection, lysogeny, and horizontal gene transfer [6, 7], affecting microbial succession, evolution, and ecosystem function [4, 8]. Thus, understanding viral ecology in WWTPs is essential for advancing ecological theory and guiding microbial community management [9].

According to community assembly theory, deterministic (e.g. host specificity and environmental filtering) and stochastic (e.g. dispersal and birth–death dynamics) processes affect viral composition, and their relative effect varies across space and time [10, 11]. These processes generate macroecological signatures, such as species abundance distributions (SADs) that distinguish common from rare taxa [12] and temporal turnover rates that reflect the succession pace [13], which together constrain viral biogeography and niche differentiation and affect bacterial community structure [14]. Furthermore, contrasting life-history strategies of virulent and temperate viruses produce distinct population dynamics and ecological niches, complicating viral assembly and virus–host interactions [15].

Virus–host interactions are key regulators of bacterial communities in complex ecosystems [16]. Given their high host specificity, viral abundance can track or even precede bacterial population shifts [17], prompting a predictive question of both theoretical and applied importance: to what extent does virome composition reflect bacterial community dynamics [18]? Integrative approaches combining host prediction, abundance correlations, and co-occurrence networks have been used to clarify these interactions in various systems (e.g. the human gut) [19]. Quantifying such interactions may reveal ecological feedbacks shaping bacterial assembly and functional trajectories, enabling viral signatures to serve as real-time indicators for microbial monitoring and control in engineered treatment systems.

Although viruses are ecologically important in WWTPs [20], they remain poorly characterized in engineered ecosystems, particularly their temporal dynamics, assembly mechanisms, and virus–host interactions. To address these gaps, we analyzed a year-long metagenomic time-series dataset from activated sludge (AS) and anaerobic treatment (AT) systems in four full-scale industrial textile WWTPs, as previously described [21]. We (i) characterized viral distribution, assembly processes, and temporal succession; (ii) examined virus–bacteria–environment relationships and virus–host coupling at community and gene levels; and (iii) evaluated the extent to which virome composition influences and predicts bacterial community dynamics. We hypothesized that viral and bacterial successions are tightly coupled, and that viral signatures are sensitive predictors of bacterial dynamics and system conditions, providing a basis for virome–informed monitoring and management of engineered ecosystems.

## Materials and methods

### Sample collection, environmental conditions, and metagenomic sequencing

Between October 2018 and October 2019, we collected 146 AS and 186 AT samples from four full-scale WWTPs (IG, TG, TV, and VNG) in Southeast Asia. All plants treated wastewater from textile mills producing similar products, except TG, which processed a mix of textile and municipal wastewater. Planktonic samples (500 ml wastewater) were collected every two weeks from the well-mixed zone of the tank and filtered through 0.22 μm membrane filters (47 mm; Durapore, Germany), whereas biofilm samples were collected monthly by scraping ~50 g of material from carrier surfaces. All samples were collected during routine morning monitoring at each plant. Both sample types were processed for metagenomic sequencing. System configurations are presented in Fig. S1, and environmental conditions, including a total of 13 parameters (e.g. pH, temperature, and chemical oxygen demand [COD] concentration), are summarized in Table S1 and detailed elsewhere [22]. All WWTPs primarily targeted COD removal, treating an average of 208.2 ± 113.3 m^3^ wastewater per day. The COD loading rate ranged from 714.0 ± 146.8 to 750.2 ± 149.3 mg/m^3^/d in IG, TV, and VNG but was lower (270.4 ± 79.7 mg/m^3^/d) in TG. In these WWTPs, AT systems served mainly as pre-treatment processes, with the majority of COD removal occurring in downstream AS systems. Accordingly, COD removal efficiencies ranged from 20.2% ± 10.6% to 65.5% ± 10.7% in AS systems and 15.9% ± 4.9% to 39.9 ± 10.8% in AT systems.

Because COD removal primarily occurred in AS systems, first-order [23] and Grau second-order [24] kinetic models were fitted to temporal COD concentration data from the AS units to estimate plant-specific COD removal kinetics. The model with a higher coefficient of determination (*R*^2^), determined using a linear model with plant as a fixed effect, was retained to estimate plant-specific COD removal kinetics. Additional details are provided in Text S1.

Genomic DNA was extracted using the DNeasy PowerSoil Kit (Qiagen, Germantown, MD, USA) following the manufacturer’s protocol. Metagenomic libraries were sequenced on the NovaSeq platform (Illumina, San Diego, CA, USA) to generate 150-bp paired-end. Each sample yielded ~14.8 million raw reads, of which ~14.3 million were retained after filtering using illumina-utils [25] (v2.4.1, default settings).

### Viral contig recovery, quality filtering, and viral operational taxonomic unit profiling

Paired-end reads from each sample were assembled using MEGAHIT [26] (v1.2.9), and contigs <1000 bp were discarded. To maximize viral recovery, VirSorter2 [27] (v2.2.3, default parameters) and DeepVirFinder [28] (v1.0; prediction score ≥ 0.5, *P* < .05) were applied. Contigs identified using either tool were merged per sample, yielding ~0.9 and 1.2 million sequences from AS and AT samples, respectively.

Quality was examined using CheckV [29] (v1.0.1, “end_to_end” mode, default settings). Rather than applying CheckV’s quality tiers based on completeness estimates, contigs were designated as high-quality if they met the following custom criteria: (i) more viral than host genes, (ii) >3000 bp, (iii) low internal repeats (k-mer frequency ≤ 1), (iv) no CheckV warnings (e.g. multiple viral regions or excessive length), and (v) ≥50% estimated completeness [30]. High-quality contigs were clustered into high-quality viral operational taxonomic units (HQ vOTUs) at 95% average nucleotide identity and 85% alignment fraction [31], with the longest contig serving as the representative.

Taxonomic classification of HQ vOTUs was performed using PhaGCN2 [32] (v2.2) with default settings, based on the latest ICTV reference database available at the time of installation (May 2024). To calculate reads per kilobase per million mapped reads (RPKM), representative sequences were indexed using BWA (v0.7.18-r1243), and reads were mapped using CoverM (v0.5.0; https://github.com/wwood/CoverM) under the following parameters: “—min-read-percent-identity 90,” “—min-covered-fraction 75,” and “—methods rpkm” [30, 33]. A HQ vOTU was considered present if >75% of its length was covered at ≥90% identity. RPKM values were normalized per sample to estimate relative abundance [34]. Viral lifestyle (temperate or virulent) was predicted using PhaTYP [35] (v1.0), retaining predictions with scores >0.5 [36].

### Intra-population genetic variation and selection analysis

Intra-population genetic variation (microdiversity) within viral communities was examined using InStrain [37] (v1.10.0). HQ vOTUs with a mean coverage of ≥5× were retained, and nucleotide diversity was calculated at positions with ≥5× coverage.

Single-nucleotide variants (SNVs) were identified using a minimum allele frequency of >0.05 and a false discovery rate of ≤1 × 10^−6^ and classified as synonymous or nonsynonymous based on gene annotations. The ratio of nonsynonymous to synonymous substitutions (pN/pS) was used to infer selection (pN/pS <1, purifying; >1, positive). Community-level nucleotide diversity and SNV density (per 1000 bp) were estimated using abundance-weighted averages.

### Metagenome-assembled genome reconstruction and virus–host prediction

High-quality representative metagenome-assembled genomes (HQ rMAGs) [21], were used to characterize bacterial community composition. Contigs were binned using MaxBin2 [38] (v2.2.7) with default settings. Dereplication of MAGs from the AS and AT systems was performed separately using dRep [39] (v2.6.2), with a primary clustering threshold of 90% Mash similarity and a secondary threshold of 99% average nucleotide identity. MAG completeness and contamination were assessed using CheckM [40] (v1.1.3) with default parameters. Only HQ rMAGs (>90% completeness, <5% contamination) were retained. In total, 145 AS and 169 AT HQ rMAGs were recovered, recruiting an average of 48.6% ± 11.5% and 64.8% ± 14.4% of total reads, respectively. Taxonomic classification of HQ rMAGs was performed using GTDB-Tk [41] (v1.5.0) with the R06-RS202 reference database. Relative abundances were estimated using the same RPKM-based approach as for HQ vOTUs.

To assess putative viral host range, we linked vOTUs to HQ rMAGs using two in silico approaches. CRISPR repeats and spacers were assembled from metagenomic reads using Crass [42] (v1.0.1) with default parameters. Spacer sequences were queried against vOTUs using BLASTn (BLAST [43] v2.16.0), retaining hits with alignment length ≥ 23 bp, nucleotide identity ≥95%, and at most one mismatch. CRISPR repeat sequences were queried against HQ rMAGs using BLASTn, retaining only exact matches (100% identity and 0 mismatches). In addition, iPHoP [44] (v1.3.3) was used to predict additional virus–host linkages using HQ rMAGs as candidate hosts, retaining predictions with confidence score ≥ 90 [18]. Temporal coupling between viral (temperate and virulent) and host (HQ rMAG) populations was examined using cross-correlation functions (CCFs) on log_10_-transformed relative abundances with the ccf21 package (v0.0.0.9) in R (v4.2.3). Significance was determined using Fisher’s z-transformed 95% confidence intervals (CIs); CCFs were significant only when the entire interval lay above or below zero [45]. Positive lags indicated that host shifts preceded viral responses, whereas negative lags indicated the reverse.

### Viral community diversity and temporal dynamics

Viral community α- and β-diversity were examined using the vegan package (v2.5.6) in R. β-diversity was calculated using Bray–Curtis dissimilarity, and compositional differences among plants were tested using permutational multivariate analysis of variance (PERMANOVA) and visualized using principal coordinates analysis (PCoA). Similarities between viral and bacterial compositions and between viral composition and environmental variables were examined using Procrustes and Mantel tests in vegan. Temporal viral dynamics within plants were evaluated using time–decay relationships [46] and halving-time estimates [47], where steeper slopes or shorter halving times indicate faster turnover. Additional details are provided in Text S1.

### Modeling species abundance distributions and community assembly processes

SADs were modeled to characterize dominance–rarity patterns and infer assembly processes in viral and bacterial communities. Four SAD models—broken-stick, log-series, Poisson-lognormal, and Zipf—were fitted using the “fitsad” function in the sads package (v0.6.3) in R*.* Maximum likelihood estimates were obtained, and predicted rank–abundance curves were generated using the “radpred” function. Model performance was evaluated using a modified coefficient of determination [48]:

$${R}^2=1-\frac{\sum{\left({\mathit{\log}}_{10}(obs)-{\mathit{\log}}_{10}(pred)\right)}^2}{\sum{\left({\mathit{\log}}_{10}(obs)-\overline{{\mathit{\log}}_{10}(obs)}\right)}^2}$$

where *obs* and *pred* represent the observed and predicted species relative abundances, respectively.

Community heterogeneity was quantified using Taylor’s power law (variance = *a ∙ mean^b^*), by regressing log_10_(mean) against log_10_(variance) of population relative abundances. The slope (*b*) indicates variability [49].

Community assembly was evaluated by comparing observed co-occurrence patterns with null expectations using the checkerboard C-score, calculated with the EcoSimR package (v0.1.0) in R. Null distributions were generated from 10 000 sequential-swap randomizations, and a standardized effect size (SES) was computed as follows [50]:

$$SES=\frac{Cscore_{observed}-{Cscore}_{null}}{SD\left({Cscore}_{null}\right)}$$

Higher observed C-scores (*Cscore_observed_*) relative to the null (*Cscore_null_*) indicate nonrandom deterministic assembly. SES values greater than 2 and less than −2 indicate species segregation and aggregation, respectively [50]. Niche partitioning among temperate and lytic viruses was examined using the Sloan neutral model [51], which relates species prevalence to relative abundance across samples [19]. Observed HQ vOTU prevalence and abundance distributions were compared with neutral expectations. HQ vOTUs outside the model’s 95% CI were considered nonneutral: those with higher prevalence but lower abundance were considered as “above neutral” (segregation), and those with higher abundance but lower prevalence as “below neutral” (aggregation). HQ vOTUs within the confidence bounds were considered neutral, consistent with stochastic assembly.

### Co-occurrence network construction and analysis

Co-occurrence analysis was performed using HQ vOTUs with the cooccur package (v1.3) in R, and only significant associations (*P* < .001) were retained [52]. Each HQ vOTU was assigned to the plant where it exhibited the highest mean relative abundance, defining plant-specific HQ vOTUs. Networks were visualized using Gephi [53] (v0.9.2). Viral network topology was characterized by calculating the module-specific participation coefficient (PM) for each HQ vOTU as follows [54]:

$${PM}_{i,m}=\frac{k_{i,m}}{k_i}$$

where *k_i,m_* is the number of edges from node *i* to module *m*, and *k_i_* is the total number of edges connected to node *i*. This metric quantifies a node’s connection proportion to each module, indicating its localized integration within the network.

To compare network topology between viral and bacterial communities, bacterial co-occurrence networks were constructed using HQ rMAGs following the same method and significance threshold as for viral networks.

Pairwise dissimilarity among viral and bacterial co-occurrence networks across the four plants was quantified using the network dissimilarity index (*β_WN_*), defined as follows [55]:

$${\beta}_{WN}=\frac{b+c}{\left(2a+b+c\right)/2}-1$$

where *a* is the number of shared edges and *b* and *c* are edges unique to each network. Higher *β_WN_* values indicate greater structural dissimilarity.

Network robustness was examined using natural connectivity [56], which measures the redundancy of alternative paths within a network:

$$\overline{\lambda}=\mathit{\ln}\left(\frac{1}{n}\ \sum_{i=1}^N{e}^{\lambda_i}\right)$$

where *λ*_i_ are the eigenvalues of the adjacency matrix, and *N* is the total number of nodes. To evaluate robustness under targeted attack, nodes were ranked by betweenness centrality and sequentially removed in descending order until 80% were eliminated. Natural connectivity was recalculated after each removal to quantify residual network resilience.

### Directional causality between viral and bacterial communities

Convergent cross mapping (CCM) was applied to infer directional causality between viral and bacterial community dynamics over time. Analyses were performed using the multispatialCCM package (v1.3) in R. For each WWTP, viral and bacterial dynamics were represented by the first two principal coordinate axes (PCoA1 and PCoA2) derived from Bray–Curtis dissimilarities. All combinations of viral and bacterial PCoA axes were tested in both causal directions: viral-to-bacterial and bacterial-to-viral. The optimal embedding dimension (E) for each time series was selected independently by maximizing short-term prediction skill using simplex projection, with a candidate range of E = 2 to 5 and a fixed time lag (τ = 1). CCM analyses were performed using 10 000 bootstrap iterations to estimate cross-map skill (ρ) across increasing library sizes (L).

Directional causality was inferred when CCM results showed positive convergence, defined as: (i) a positive relationship between ρ and L (ρ_slope_ > 0), and (ii) a positive ρ at the maximum library size (ρ_max_) [57, 58]. In cases where bidirectional convergence was detected, the relative strength of influence was assessed by comparing ρ_max_ values, with the direction showing the higher ρ_max_ interpreted as having stronger casual inference [57].

### Generalized linear mixed models and structural equation modeling

To evaluate how temporal variation in COD-related variables affected viral and bacterial community structure in AS systems, generalized linear mixed models (GLMMs) and generalized additive mixed models (GAMMs) were used. Predictor variables included reactor COD concentration, COD loading, COD removal efficiency, COD removal rate, and sample type (planktonic or biofilm). Multicollinearity was evaluated using Pearson’s correlation (*r* < 0.5) and variance inflation factors (<2); collinear variables were excluded. COD concentration, loading, and removal efficiency were retained as fixed effects, with plant as a random effect. GLMMs were fitted using glmmTMB (v1.1.11) and GAMMs using gamm (v0.2–7) in R. Model performance was evaluated using the corrected Akaike information criterion (AIC) in MuMIn (v1.48.4) in R, with all candidate models fitted using maximum likelihood for AIC-based comparisons. To investigate the direct and indirect effects of COD variables on viral and bacterial communities, piecewise structural equation modeling (SEM) was performed using the piecewiseSEM package (v2.3.0.1) in R.

### Deep learning prediction of bacterial communities from viromes

To predict bacterial community composition from viral dynamics in AS systems, a deep learning approach was used. To reduce dimensionality and capture dominant variation patterns in high-dimensional relative abundance data, singular value decomposition (SVD) was performed on viral and bacterial compositions [59]. This step yielded orthogonal components ranked by explained variance. This analysis (i) predicted major bacterial SVD components from viral components using a neural network and (ii) reconstructed bacterial taxon-level abundances from predicted components.

A feedforward neural network was constructed for component-level prediction. The input layer contained one node per viral SVD component explaining ~80% of total viral variance. The output layer included one node per bacterial SVD component explaining a comparable proportion of bacterial variance. A single hidden layer was used, with neurons *H* ∈ {1, 2, …, 33} tuned in unit steps. The model was trained on 80% of AS samples and tested on the remaining 20% samples. Performance for each output component was evaluated using the *R*^2^, mean absolute error (MAE), and root mean squared error (RMSE). To account for the contribution of each component to the total variance, performance metrics were normalized and weighed as follows [60]:

$${v}_i=\frac{\sigma_i^2}{\sum_{i=1}^k{\sigma}_i^2}$$

where σ_i_ is the singular value of the *i*-th bacterial component and ν_i_ is its corresponding weight.

Weighted metrics were calculated as

$${M}_w=\sum_{i=1}^{\mathrm{k}}{v}_i\bullet{M}_i$$

for each performance metric *M* ∈ {*R*^2^, MAE, RMSE}.

To enable metric aggregation, each weighted metric was standardized as a z-score:

$${z}_{R^2}=z\ \left({R}_w^2\right),{z}_{MAE}=z\left(-{MAE}_w\right),{z}_{RMSE}=\left(-{RMSE}_w\right)$$

An equally weighted composite performance score was then computed as

$${z}_{total}=0.33\bullet{z}_{R^2}+0.33\bullet{z}_{MAE}+0.33\bullet{z}_{RMSE}$$

The hidden-layer size *H* that maximized z_total_ was selected, and the final model was retrained using the full training dataset.

To reconstruct bacterial taxon-level relative abundances, a separate linear regression model was fitted for each taxon using the training data [3]:

$${Y}_j={\beta}_{0j}+\sum_{i=1}^k{\beta}_{ij}\bullet{SVD}_i+{\epsilon}_j$$

where *Y_j_* is the log-transformed relative abundance of taxon *j, SVD_i_* is the *i*-th bacterial component, *β_ij_* is the regression coefficient, *β_0j_* is the intercept, and ${\epsilon}_j$is the residual error.

Using predicted SVD values (*pSVD_i_*), predicted log-abundances (*pY_j_*) were computed as

$${pY}_j={\beta}_{0j}+\sum_{i=1}^k{\beta}_{ij}\bullet{pSVD}_i$$

Predicted relative abundances were obtained by inverse transformation:

$${pAbundance}_j=\max \left(0,\exp \left({pY}_j\right)-1\right)$$

Model performance on the test set was evaluated by comparing predicted and observed bacterial SVD components using *R*^2^. A global taxon-level *R*^2^ was also calculated by comparing predicted and observed relative abundances.

A CI framework was used to classify reconstructed taxon-level abundances as well or poorly predicted. For each taxon in each sample, predictions within ±20% of observed values (80% CI) were labeled high accuracy; all others were labeled as low accuracy [61]. When observed abundance was zero, only exact matches (predicted abundance = 0) were classified as high accuracy, whereas any non-zero prediction was classified as low accuracy. For each taxon across samples, it was classified as high accuracy if ≥50% of its sample-wise predictions (≥15 of 30 samples) were accurate. For sample-level accuracy, a sample was considered high accuracy if ≥50% of its taxa (≥73 of 145) were accurately predicted.

To contextualize predictive accuracy within ecological interactions, both virus–host interactions and bacteria–bacteria associations were analyzed. For virus–host interactions, taxon-level scores represented the total number of viral populations linked to each taxon. Sample-level scores were obtained by weighting each virus–host pair by the host’s relative abundance and summing values across all pairs. For bacteria–bacteria associations, the same approach was applied. Taxon-level scores reflected total pairwise co-occurrences with other taxa, whereas sample-level scores were calculated by weighting each co-occurring pair by the product of their relative abundances and summing across all present pairs in each sample.

### Statistical analysis

Univariate metrics (e.g. α-diversity, nucleotide diversity, neutral model residuals, and participation coefficient) were compared using Mann–Whitney *U* tests (two groups) or Kruskal–Wallis tests (more than two) in the stats package (v4.1.2) in R. Significance was set at *P* < .05.

## Results

### Plant-specific virome composition in WWTPs

To investigate temporal viral dynamics, samples were collected every two weeks from AS and AT systems of four full-scale textile WWTPs over 1 year (Fig. S1). Viral identification using two independent tools (VirSorter2 and DeepVirFinder) recovered an average of 6240 ± 1699 and 6576 ± 2034 viral contigs per sample from AS and AT systems, respectively. Following contig-level filtering based on specific quality criteria [30], a total of 1654 and 2461 high-quality viral contigs were retained from AS and AT systems, respectively. Clustering at 95% average nucleotide identity and 85% alignment fraction yielded 1046 HQ vOTUs from AS systems and 1386 from AT systems (Tables S2 and S3), representing 0.7% ± 0.4% and 1.1% ± 0.6% of total metagenomic reads per sample, respectively (Fig. S2A), consistent with previous studies [62, 63].

**Figure 1:**
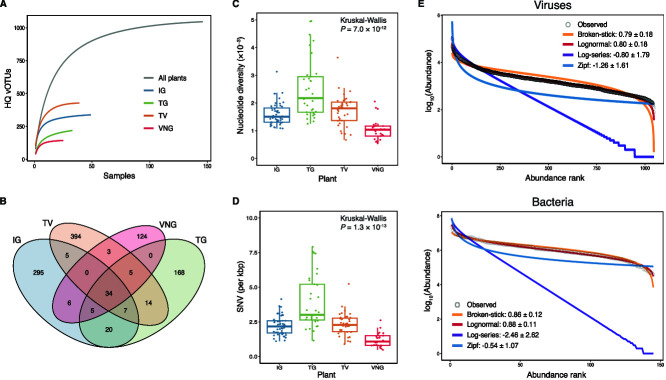
Distribution and diversity of HQ vOTUs in AS systems. (A) Accumulation curves showing the number of HQ vOTUs as a function of sample number for individual plants and all plants combined. (B) Number of HQ vOTUs shared among four plants. (C) Abundance-weighted nucleotide diversity of viral communities across plants. (D) Abundance-weighted single-nucleotide variant (SNV) density of viral communities across plants. (E) Abundance distributions of viral and bacterial communities fitted to four species-abundance models, namely lognormal, log-series, broken-stick, and Zipf, with corresponding *R*^2^ values indicating model fit.

Rarefaction curves plateaued across all plant- and system-level samples, indicating sufficient sequencing depth to capture most viral diversity (Figs. 1A and S3A). Both systems exhibited strong plant-specific virome signatures, with only 34 HQ vOTUs in AS and 32 in AT shared across all four plants (Figs. 1B and S3B). Taxonomic annotation revealed that most HQ vOTUs belonged to *Caudoviricetes*—90.8% in AS systems and 96.9% in AT systems (Fig. S4)—indicating the dominance of tailed bacteriophages in WWTP viromes [64]. Most remained unclassified at lower taxonomic levels.

Lifestyle predictions indicated a predominance of virulent viruses, comprising 53.6% of AS and 57.4% of AT HQ vOTUs (Fig. S5). Among temperate viruses, 5.9% in AS systems and 3.4% in AT systems were identified as integrated prophages based on CheckV. Consecutive-month community dissimilarity analyses revealed significantly higher turnover of free temperate viruses compared to prophages in AT systems (0.55 ± 0.16 vs. 0.43 ± 0.15; Mann–Whitney test, *P* = 2.0 × 10^−4^), whereas this difference was not statistically significant in AS systems (0.53 ± 0.13 vs. 0.50 ± 0.25; Mann–Whitney test, *P* = .07; Fig. S6). The ratio of virulent to temperate viral relative abundances fluctuated over time within individual plants (Fig. S7). However, mean ratios remained consistently below 1 in all AS (0.61 ± 0.21–0.90 ± 0.24) and AT (0.37 ± 0.08–0.48 ± 0.17) systems, with the exception of the AS system at plant VNG (1.23 ± 1.15), indicating a general predominance of temperate viruses over time.

### Stronger plant-specific structuring and network modularity in viral communities

In both AS and AT systems, viral α-diversity (Shannon index) significantly differed among plants (Kruskal–Wallis test, both *P* < 2.2 × 10^−16^), with the highest diversity at IG (Fig. S2B). PCoA with PERMANOVA showed that community composition was primarily structured by plant, with stronger clustering in AS (pseudo-*F* = 28.4, *P* = .001) and AT (pseudo-*F* = 42.7, *P* = .001) systems than by sample type (planktonic vs. biofilm; AS: pseudo-*F* = 3.2, *P* = .002; AT: pseudo-*F* = 5.3, *P* = .001; Fig. S8). Compared with bacterial communities based on HQ rMAGs (AS: pseudo-*F* = 21.5; AT: pseudo-*F* = 22.9; both *P* = .001 [21]), viral communities exhibited greater between-plant divergence. Seasonal turnover patterns revealed significantly higher within-season dissimilarity in viral communities (AS: 0.30 ± 0.17–0.78 ± 0.21; AT: 0.19 ± 0.05–0.78 ± 0.31) than in bacterial communities (AS: 0.12 ± 0.03–0.67 ± 0.33; AT: 0.11 ± 0.05–0.68 ± 0.32; Mann–Whitney test, both *P* < .001) in both systems (Fig. S9). In general, viral and bacterial community dissimilarities were lower in winter than in other seasons, with the exception of plant TG and the AT system of plant TV.

Viral microdiversity across AS and AT systems was examined using nucleotide diversity, SNV density, and gene-level pN/pS ratios (Tables S4 and S5). Both abundance-weighted nucleotide diversity and SNV density significantly differed among plants in both systems (Kruskal–Wallis test, all *P* < .001; Figs. 1C, D, S3C and D). Plant TG consistently exhibited the highest nucleotide diversity (AS: 2.5 × 10^−3^ ± 1.1 × 10^−3^; AT: 4.9 × 10^−3^ ± 1.2 × 10^−3^) and SNV density (AS: 3.9 ± 1.9; AT: 7.1 ± 1.6). Among genes with synonymous or nonsynonymous SNVs, most had a pN/pS < 1 (AS: 60490; AT: 150277), indicating widespread purifying selection, where deleterious nonsynonymous mutations are removed to maintain protein function and gene stability [65] (Fig. S10). A smaller subset (AS: 8754; AT: 20839) had a pN/pS > 1, suggesting localized positive selection and adaptive evolution acting on specific viral genes [66].

Viral co-occurrence networks exhibited strong modularity, indicating plant-specific niche differentiation (Figs. S11A and S12A). Positive associations were significantly stronger (Kruskal–Wallis test, *P* < 2.2 × 10^−16^) among HQ vOTUs dominant within the same plant (AS: PM = 0.44; AT: PM = 0.42) than between plants (both AS and AT: PM = 0.01), suggesting niche-specific interactions. Conversely, negative associations were significantly stronger (Kruskal–Wallis test, *P* < 2.2 × 10^−16^) between HQ vOTUs dominant in different plants (AS: PM = 0.02; AT: PM = 0.03) than within plants (AS: PM = 4.0 × 10^−4^; AT: PM = 5.4 × 10^−5^), suggesting competitive interactions between plant-specific viral communities (Figs. S11B and S12B).

Bacterial co-occurrence networks constructed using HQ rMAGs comprised 1373 positive and 1027 negative correlations in AS systems and 1843 positive and 1404 negative correlations in AT systems (Tables S6 and S7). Sequential node removal showed that viral networks were more stable than the bacterial network in AS systems (network slope: all viruses = −0.002; bacteria = −0.004; all *P* < .001), whereas robustness was comparable in AT systems (all network slopes = −0.004; all *P* < .001; Figs. S11C and S12C). Network dissimilarity analysis revealed higher variability among viral networks across plants (AS: *β_WN_* = 0.97–1.0; AT: *β_WN_* = 0.98–0.99) than bacterial networks (AS: *β_WN_* = 0.74–0.97; AT: *β_WN_* = 0.76–0.97; Table S8), consistent with PCoA results showing stronger plant-specific clustering in viral communities.

### Abundance patterns and temporal turnover of viral and bacterial communities

SAD models revealed that the lognormal model, characterized by a log-scale unimodal distribution, best described both bacterial (*R*^2^ = 0.88 ± 0.11 in AS; *R*^2^ = 0.76 ± 0.17 in AT) and viral communities (*R*^2^ = 0.80 ± 0.18 in AS; *R*^2^ = 0.76 ± 0.20 in AT; Figs. 1E and S3E), indicating dominance by few abundant and many rare taxa. Relative abundance patterns followed Taylor’s power law, exhibiting a positive linear mean–variance relationship (Fig. S13). Larger Taylor exponents for viruses (*b* = 1.43 in AS; 1.41 in AT) than bacteria (*b* = 1.28 in AS; 1.34 in AT) suggested greater spatiotemporal variability in viral communities.

**Figure 2:**
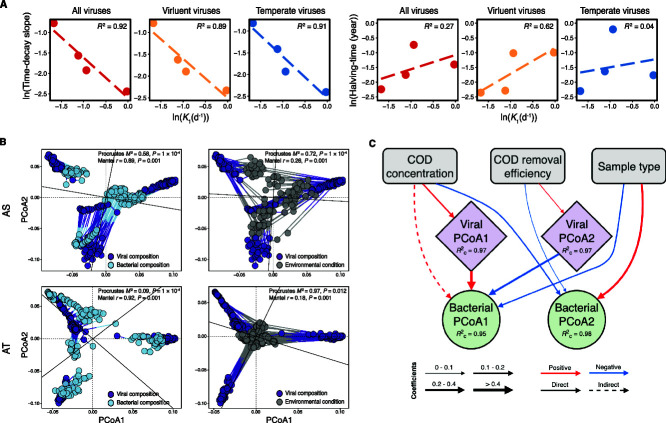
Relationships between environmental parameters, bacterial communities, and viral communities in AS systems. (A) Correlation between the first-order COD removal rate coefficient (*K_1_*) and the time-decay slope (*c*) or halving-time (HT) of viral community composition. *R*^2^ values are presented for each correlation. (B) Procrustes and mantel tests were performed to examine correspondence between viral community composition and either environmental parameters (including all 13 measured variables) or bacterial community composition. (C) Structural equation modeling illustrating direct and indirect effects of COD-related parameters on viral and bacterial community composition.

Viral community turnovers, evaluated using time–decay slopes and halving-times, exhibited significant correlations between community dissimilarity and time in both AS and AT systems, indicating ongoing temporal turnover. Slopes were steeper in AS (0.09–0.46) than AT (0.04–0.24) systems, suggesting faster turnover (Figs. S14 and S15). Within plants, virulent and temperate viruses exhibited similar slopes (AS: 0.10–0.45 vs. 0.10–0.44; AT: 0.05–0.23 vs. 0.04–0.23), but virulent viruses consistently showed shorter halving-times, reflecting more rapid shifts (Figs. S16 and S17). Viral halving-times were also shorter than those previously estimated for bacterial communities [21] in the same systems. Correlations between first-order COD removal rate coefficients and viral time-decay slopes or halving-times revealed a strong association between viral turnover and treatment performance in AS systems (Fig. 2A). Time–decay slopes were highly correlated with first-order COD removal rates (*R*^2^ = 0.89–0.92), whereas halving-times showed weak to moderate correlations (*R*^2^ = 0.04–0.62), highlighting the coupling between viral temporal dynamics and system function.

### Nonneutral assembly of viral communities across lifestyles

**Figure 3:**
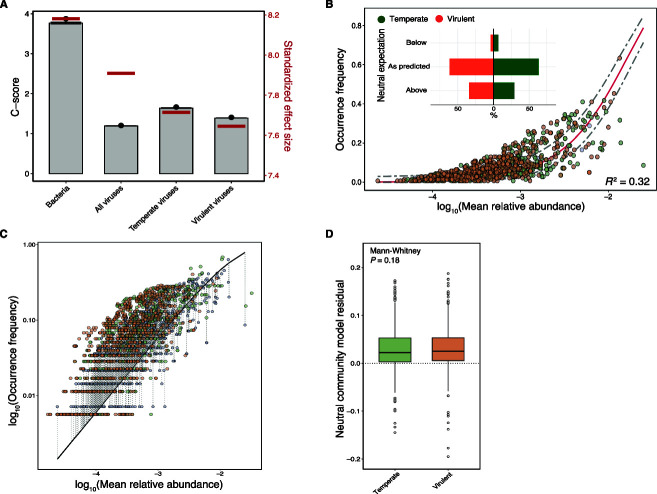
Ecological processes affecting viral community assembly in AS systems. (A) C-score analysis was performed using null models for bacterial communities and all, temperate, and virulent viruses. Dots indicate observed C-scores; bars show simulated C-scores with 95% CIs. Standardized effect sizes less than −2 or greater than 2 indicate significant aggregation and segregation, respectively. (B) Fit of the Sloan neutral model to viral communities. Each point represents a HQ vOTU, colored by predicted lifestyle (temperate or virulent). The solid line shows the neutral model prediction; dashed lines indicate the 95% confidence interval. *R*^2^ denotes model fit. The inset bar chart shows the number of HQ vOTUs falling below, within, or above neutral expectations. (C) Residuals from the neutral model based on log-transformed HQ vOTU prevalence. (D) Comparison of neutral model residuals between temperate and virulent HQ vOTUs.

Community assembly mechanisms evaluated using the C-score metric revealed significantly greater segregation than expected under null models (all SES > 2; AS: 7.7–8.2, AT: 8.6–9.0; Figs. 3A and S18A), indicating nonrandom structures in both viral and bacterial communities. Across all plants, SES values consistently exceeded 2 in AS and AT systems, supporting the dominance of deterministic processes over time (Fig. S19).

Stochastic assembly was evaluated using the Sloan neutral model, which showed a poor fit for viral communities in AS (*R*^2^ = 0.32) and AT (*R*^2^ = 0.05) systems (Figs. 3B and S18B). Many HQ vOTUs deviated from neutral expectations, particularly virulent viruses. In AS systems, 34.4% of virulent and 29.5% of temperate HQ vOTUs lay above neutral expectations, whereas 3.9% and 6.9%, respectively, fell below. In AT systems, 42.3% of virulent and 33.5% of temperate HQ vOTUs exceeded predicted prevalence. Residuals (observed minus predicted prevalence) were positive for both lifestyles—temperate (AS: 0.023 ± 0.07, AT: 0.024 ± 0.08) and virulent (AS: 0.028 ± 0.05; AT: 0.036 ± 0.06)—indicating higher-than-expected prevalence (Figs. 3C and S18C). Residuals did not significantly differ between lifestyles in AS systems (Mann–Whitney test, *P* = .18) but were significantly higher for virulent viruses in AT systems (*P* = 9.8 × 10^−3^; Figs. 3D and S18D). These results suggest that under anaerobic conditions, virulent viruses deviate more from neutral assembly and become disproportionately widespread than temperate viruses.

### Viruses mediate the link between environmental conditions and host communities

To further investigate virus–bacteria–environment relationships, similarities between bacterial community composition and either viral communities or environmental parameters (based on 13 measured variables) were analyzed using Procrustes and Mantel tests (Fig. 2B). In both AS and AT systems, viral–bacterial similarity (AS: *M*^2^ = 0.58, *r* = 0.89; AT: *M*^2^ = 0.09, *r* = 0.92; all *P* < .05) exceeded viral–environmental similarity (AS: *M*^2^ = 0.72, *r* = 0.26; AT: *M*^2^ = 0.97, *r* = 0.18; all *P* < .05), indicating stronger viral coupling with bacterial composition than with environmental factors.

Given the central role of AS systems in COD removal, virus–bacteria–environment relationships were examined. CCM was first applied to assess directional causality between viral and bacterial community dynamics over time. In most plants, planktonic communities showed positive CCM convergence (ρ_slope_ > 0) in both directions (virus-to-bacteria and bacteria-to-virus). The resulting ρ_max_ values were similar between directions (virus-to-bacteria: 0.09 ± 0.13 to 0.96 ± 0.01; bacteria-to-virus: 0.07 ± 0.14 to 0.96 ± 0.01), with differences generally small and within overlapping 95% CIs (Fig. S20A). Overall, this pattern is consistent with bidirectional coupling rather than a predominant unidirectional effect. By contrast, CCM convergence was less frequent in biofilm communities (Fig. S20B); when present, it occurred in fewer plants and was more often detected only in the bacteria-to-virus direction.

To further evaluate the extent to which virome composition influences bacterial community dynamics, virus–bacteria–environment relationships were quantified using GLMMs, GAMMs, and piecewise SEM. GLMMs consistently outperformed GAMMs based on lower AIC values, indicating that linear mixed models provided a better fit to the data than additive models with non-linear smooth terms (Table S9). Therefore, GLMMs were used to evaluate the effects of COD concentration, loading, and removal efficiency along with sample type on viral and bacterial α- and β-diversity (PCoA1 and PCoA2). GLMMs showed that COD concentration and removal efficiency significantly affected viral and bacterial composition (Fig. S21). SEM further clarified the direct and indirect effects of COD-related parameters on the compositions of viral and bacterial communities (Fig. 2C): COD concentration positively affected viral PCoA1 (standardized coefficient = 0.15), whereas COD removal efficiency positively affected viral PCoA2 (0.08). Viral composition, in turn, strongly shaped bacterial communities (PCoA1: 0.67, PCoA2: −0.36), revealing an indirect pathway through which COD concentration affected bacterial structures via viruses (net indirect effect: 0.10). Together, these results highlight viruses as ecological intermediaries mediating COD-driven environmental effects on bacterial community dynamics.

### Virus–host coupling enables predictive modeling of bacterial composition

**Figure 4:**
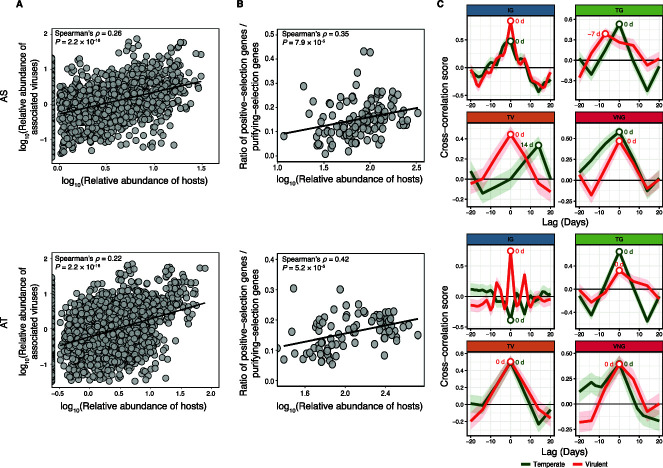
Virus–host coupling and synchrony in AS and AT systems. (A) Relationship between the relative abundances of all detected hosts and their associated viruses, evaluated using Spearman’s rank correlation. (B) Relationship between host relative abundance and viral selection signals. Associations were evaluated using Spearman’s rank correlation. (C) Cross-correlation analysis was performed between viral and associated host bacterial abundances. Lines represent cross-correlation scores (green for temperate viruses and orange for virulent viruses). The x-axis shows time lag in days; positive values indicate the viruses lag behind the hosts, whereas negative values indicate the viruses precede the hosts. Peaks at lag = 0 days suggest synchronous virus–host dynamics. Shaded ribbons indicate 95% CIs. Circles denote peak cross-correlation values and their corresponding time lags.

Virus–host mapping identified 529 predicted interactions in AS systems and 759 in AT systems (Fig. S22). CRISPR spacer matching accounted for 490 (AS) and 626 (AT) linkages, with iPHoP contributing an additional 39 (AS) and 133 (AT) pairs (Tables S10 and S11); thus, the interaction set was driven primarily by CRISPR-based linkages. Putative hosts were mainly affiliated with *Proteobacteria, Bacteroidota, Chloroflexi*, and *Acidobacteriota* (Tables S10 and S11)*.* At the plant level, bacterial phylum abundance correlated with the summed abundance of associated viruses, although correlation strength varied by phylum and plant (Fig. S23). For instance, in AS systems, *Planctomycetota* showed a strong correlation in plant IG (ρ = 0.76, *P* = 1.3 × 10^−7^) and moderate correlations in TV (ρ = 0.53, *P* = 5.8 × 10^−4^) and VNG (ρ = 0.54, *P* = 5.8 × 10^−3^). Across all virus–host pairs, correlations were significant in both systems (AS: ρ = 0.26; AT: ρ = 0.22; both *P* = 2.2 × 10^−16^), indicating consistent virus–host coupling (Fig. 4A). Moreover, the ratio of positively to purifying-selected genes correlated with host abundance (AS: Spearman’s ρ = 0.35, *P* = 7.9 × 10^−5^; AT: ρ = 0.42, *P* = 5.2 × 10^−5^; Fig. 4B).

CCF analysis revealed strong temporal synchrony between viral and host communities in both systems (Fig. 4C). In AS systems, temperate and virulent viruses and their putative hosts were largely synchronous across all plants, with cross-correlation peaks predominantly at zero-day lag (CCF = 0.44–0.84). In some plants, peak synchrony occurred at non-zero lags, with temperate viruses in TV peaking at +14 days (CCF = 0.33) and virulent viruses in TG peaking at −7 days lag (CCF = 0.39). In AT systems, both lifestyles also showed synchrony with their putative hosts across all plants (CCF = 0.32–0.75), although temperate viruses in IG were negatively associated with host dynamics (CCF = −0.39). Overall, these results indicate largely synchronous and coupled virus–host temporal dynamics in both systems.

**Figure 5:**
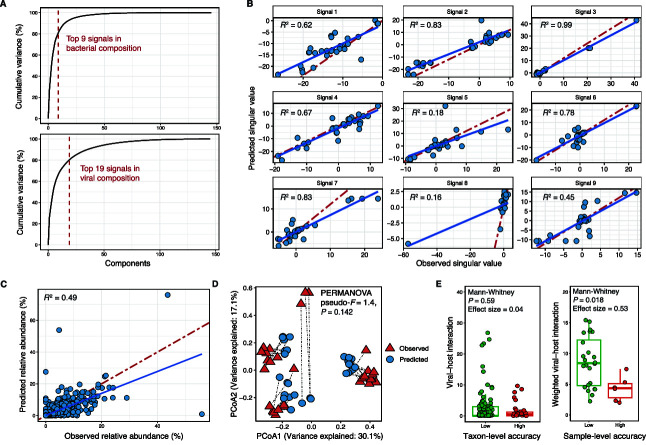
Predicting bacterial taxa from viral community composition in AS) systems. (A) Selection of top-ranked singular value decomposition (SVD) components explaining more than 80% of variance in bacterial (nine components) and viral (19 components) community composition. (B) Observed versus predicted SVD components for viral and bacterial communities determined using the optimized neural network with 20 hidden units on the testing set. The red line indicates the 1:1 reference, and the black line shows the regression fit. (C) Observed versus predicted relative abundances of bacterial taxa (HQ rMAGs) obtained using the optimized model. Each point represents one HQ rMAG in a sample; red and black lines indicate the 1:1 reference and regression fit, respectively. (D) Principal coordinates analysis of bray–Curtis dissimilarities between observed and predicted microbial communities, based on testing sets. Group differences (observed vs. predicted) were assessed using PERMANOVA. (E) Virus–host interactions at the taxon and sample levels. At the taxon level, interaction scores were summed per taxon, whereas at the sample level, scores were weighted by host relative abundance and summed across taxa. Statistical comparisons were performed using the Mann–Whitney test, with corresponding effect sizes reported.

To evaluate whether viral dynamics can predict bacterial community composition, neural networks were trained on AS system data. Dimensionality reduction via SVD identified latent components capturing dominant variation patterns. The top nine bacterial and 19 viral components (~80% cumulative variance explained; Fig. 5A) were used to train neural networks of varying architectures. The best model (20 hidden units) showed strong predictive performance on the training set (weighted *R*^2^ = 0.7 ± 0.1; weighted MAE = 1.3 ± 0.3; weighted RMSE = 2.1 ± 1.3; total score = 2.0; Fig. S24) and high prediction accuracy on the testing set (average *R*^2^ = 0.61; Fig. 5B).

Predicted SVD signals were back-transformed into taxon-level bacterial abundances using linear regression models trained on log-transformed relative abundance data. This reverse mapping showed good agreement with observed values, with a global *R*^2^ of ~0.49 at the taxon level (Fig. 5C), indicating that viral dynamics encode predictive signals of bacterial composition and reflect strong virus–host coupling in engineered ecosystems. PCoA of community dissimilarities revealed no significant difference between observed and predicted community compositions at the sample level (pseudo-F = 1.4, *P* = .142; Fig. 5D).

Prediction accuracy, evaluated using a ± 20% confidence interval threshold, classified 1743 of 4350 taxon-level predictions as high accuracy and 2607 as low accuracy. At the taxon level, neither virus–host interaction scores (high: 1.6 ± 2.8, low: 4.3 ± 10.9, *P* = .59, effect size = 0.04) nor bacteria–bacteria association scores (high: 17.9 ± 14.9, low: 16.2 ± 17.4, *P* = .27, effect size = 0.09) differed significantly between prediction accuracy groups (Figs. 5E and S25A). However, at the sample level, high-accuracy samples had significantly lower abundance-weighted virus–host interaction scores (high: 4.2 ± 1.9, low: 8.8 ± 4.1; *P* = .018, effect size = 0.53) and significantly higher abundance-weighted bacteria–bacteria association scores (high: 0.35 ± 0.05, low: 0.28 ± 0.07; *P* = .025, effect size = 0.41) than low-accuracy samples (Figs. 5E and S25A). Treatment performance, measured by the first-order COD removal rate constant, also differed significantly between prediction accuracy groups (high: *K_1_* = 0.3 ± 0.4, low: *K_1_* = 0.9 ± 0.5, *P* = .005, effect size = 0.51; Fig. S25B). Together, these findings suggest that virus–host interactions, bacteria–bacteria associations, and process performance are each associated with sample-level prediction accuracy. Virus–host abundance correlations were positive and similar in both groups (high: ρ = 0.25; low: ρ = 0.24; both *P* < .01; Fig. S26). Overall, bacterial composition prediction accuracy was most strongly associated with virus–host interactions, followed by treatment metrics and bacterial associations. Bacterial taxa with complex, dynamic virus–host pairings were less predictable, whereas communities with moderate virus–host coupling yielded more accurate predictions.

## Discussion

A central goal of microbial ecology is to elucidate mechanisms underlying microbial distribution, community assembly, and temporal turnover [67]. Although viral ecology has been increasingly studied in natural environments, such as soils, freshwater, and oceans [68–70], insights from engineered systems remain limited. Biological WWTPs provide valuable models for exploring viral and bacterial ecology, offering dense microbial communities, controlled operational parameters, and well-defined environmental gradients [71]. Using time-series metagenomics from four full-scale industrial WWTPs, this study advances the understanding of viral dynamics and identifies key drivers of viral distribution, assembly, turnover, and virus–host interactions.

Significant spatial heterogeneity in viral community composition was observed, with low HQ vOTU overlap and distinct clustering by treatment plant. This pattern aligns with the findings of a global AS virome survey indicating that geographic and environmental factors strongly affect viral diversity [4]. Viral genetic profiles also varied across plants, probably reflecting localized evolutionary pressures from plant-specific environmental and host conditions [72]. Plant TG, which treated both textile and nutrient-rich municipal wastewater, exhibited the highest viral diversity, consistent with previously reported elevated bacterial diversity and growth rates [21]. The broader and more heterogeneous host community in TG likely expanded viral niche space [73] and promoted viral gene flow through more frequent host switching and co-infection [74]. Increased host proliferation and host breadth probably increased virus–host encounters and replication cycles, promoting viral diversification [72, 75]. These results indicate that deterministic processes, particularly environmental filtering and host specificity, structure viral assemblages in wastewater treatment systems.

Temporal analyses revealed rapid viral community turnover, supported by consistent time–decay relationships and short halving times within individual plants. Viral communities shifted faster than bacterial communities, probably due to their greater sensitivity to environmental fluctuations and dependence on host dynamics [18]. Virulent viruses consistently had shorter halving times than temperate viruses, consistent with their life-history strategies. Virulent types rely on lytic replication, enabling swift responses to host and environmental changes, whereas temperate viruses turn over more slowly due to their stable, latent lifestyle [76]. Compared with AS systems, the higher turnover of free temperate viruses relative to integrated prophages in AT systems suggests a clearer separation between lytic and lysogenic dynamics, potentially reflecting stronger environmental fluctuations, coupled with stress-driven prophage induction under higher loading conditions [77, 78]. Viral turnover also correlated strongly with COD removal kinetics, highlighting the potential of viral communities as sensitive microbial indicators of treatment performance in engineered ecosystems.

Species abundance distributions for viral and bacterial communities were both best fit by lognormal models, indicating that most taxa persist at intermediate abundances under selective pressures, with few consistently rare or dominant [48]. Null model analysis (SES > 2) suggested a strong deterministic influence on viral community assembly [50]. Likewise, poor fits to the Sloan neutral model for both temperate and virulent viruses indicated that nonrandom processes affected community structure, reflecting local ecological selection instead of stochastic drift [19].

Our co-occurrence network analysis revealed key features of viral community organization, including interaction patterns, niche overlap, and potential resilience to disturbance [79]. Viral networks exhibited strong plant-specific modularity, consistent with niche partitioning and local adaptation [80]. Compared with bacterial networks, viral networks showed greater structural stability and higher topological dissimilarity across plants. This stability probably results from two factors: (i) tight ecological coupling with hosts, constraining viral niche breadth, and (ii) consistent environmental filtering selecting plant-specific assemblages [81]. Such modularity may buffer viral communities against perturbations [82], enhancing resilience and highlighting their potential as sensitive indicators of ecological stability in engineered environments.

Virus–host interactions strongly affect virome ecology and bacterial community dynamics, acting as ecological filters that restructure bacterial assemblages and shape successional trajectories [14]. The apparent bidirectional coupling between viral and bacterial dynamics, together with the faster turnover of viral communities, supports the potential use of viral signals as predictors of shifts in bacterial community composition. Correlation and synchrony analyses revealed significant viral–host associations across treatment plants, with minimal temporal lags for both temperate and virulent viruses. These patterns indicate that viral abundances closely track host dynamics, supporting models of co-evolution and feedback regulation [14]. For temperate viruses, zero-lag synchrony aligns with piggyback-the-winner dynamics, where lysogeny is favored at high host densities. Conversely, the lack of predator–prey oscillations among virulent viruses may reflect limited temporal resolution, which could obscure kill-the-winner dynamics [83]. At the gene level, positive correlations between host abundance and the ratio of positively to purifyingly selected viral genes indicate tight coupling of viral evolution to host dynamics. Higher host abundance corresponded with stronger signals of positive selection, consistent with intensified virus–host interactions [75], whereas lower abundance aligned with dominant purifying selection, suggesting genome conservation under reduced selective pressure [72]. Together, these findings highlight viruses as active biotic drivers of bacterial community structure.

Building on evidence of host-linked viral dynamics at both community and genomic levels, the predictive relationship between virome and bacterial composition further underscores the ecological importance of virus–host interactions. Our findings indicate that virome signatures contained sufficient information to approximate bacterial composition at both the taxon and sample levels, with the highest predictive performance in taxa or samples exhibiting moderate virus–host interaction complexity. Conversely, highly interconnected interactions reduced model accuracy, suggesting a trade-off between ecological complexity and predictability [84].

Although viromes comprised a small fraction of total metagenomic reads, they captured substantial variation in bacterial community structure. These insights into virus–host coupling have important implications for managing engineered microbial ecosystems. In such systems, operators typically adjust bottom-up drivers (e.g. organic loading, nutrient levels, temperature), whereas viral infections impose top-down control on microbial community structure [85]. Even under semi-controlled conditions, virus–host interactions can reshape bacterial dynamics and, at times, shift regulation from bottom-up to top-down [85, 86]. In our time series, viral turnover covaried with process kinetics and accurately predicted bacterial composition, suggesting that routine virome monitoring could provide early warning of bacterial community shifts and inform set-point adjustments to enhance system stability and performance. These findings support the integration of virome analytics into operational workflows. Although virus-based interventions may eventually allow targeted microbial control, such strategies are still in early development. In the meantime, virome monitoring conducted at appropriate intervals during stable operation, and more frequently during disturbances, can function as a predictive tool to complement existing control strategies by enabling earlier detection and informed decision-making.

Although this study provides key insights into virus–bacterium dynamics and their links to treatment performance, several limitations should be acknowledged. First, bulk metagenomics primarily captured double-stranded DNA viruses and likely underrepresents single-stranded DNA and RNA viruses. Integrating viral particle–enriched viromics with metatranscriptomics could expand detection and provide functional insights into viral activity in engineered systems. Second, spatial and temporal resolution was limited to a few WWTPs sampled every two weeks. Broader geographic coverage and higher sampling frequency would improve detection of short-lag virus–host interactions, strengthen the reliability of causal inference, and clarify relationships between viral turnover and treatment kinetics. Third, virus–host interactions and co-occurrence networks were inferred computationally; experimental validation through cultivation or perturbation studies is needed to confirm assembly mechanisms and quantify interaction strength.

In summary, this study advances the ecological understanding of viromes in engineered ecosystems by revealing how deterministic selection, viral life-history strategies, and host coupling affect viral distribution and turnover. Viral composition and intra-population genetic variation were plant-specific and primarily structured by environmental filtering and host specificity, with viromes turning over faster than bacterial communities. Network analysis revealed robust viral ecological architecture, and predictive modeling demonstrated that viral signatures can forecast bacterial composition. Collectively, these findings position viruses as both ecological drivers and indicators in WWTPs. By linking viral turnover and composition to host community dynamics, our framework enables prediction-guided management and encourages the exploration of targeted virus-based interventions. This integration offers a pathway toward real-time steering of microbial communities for optimal system performance.

## Supplementary Material

Supplementary_material_wrag120

## Data Availability

Raw sequencing data have been deposited in the NCBI Sequence Read Archive under BioProject accession number PRJNA996624. HQ rMAGs and HQ vOTUs are available in the FigShare repository at http://dx.doi.org/10.6084/m9.figshare.24623736 and http://dx.doi.org/10.6084/m9.figshare.29987518, respectively. Additional data supporting the findings of this study are available from the corresponding author upon reasonable request.
