# Supplementary material for: Host-linked virome assembly and turnover predict bacterial community structure in wastewater treatment systems
Source: ISME J. 2026 May 12;20(1):wrag120. doi: 10.1093/ismejo/wrag120 (PMC13273578; doi:10.1093/ismejo/wrag120)
Supplement: Supplementary_material_wrag120 [file Supplementary_material_wrag120.zip › Combine_SI_submit.pdf]

Supplementary Information for

Host-Linked Virome Assembly and Turnover Predict Bacterial Community Structure in Wastewater Treatment Systems

Jinjin Yu,[1] Siang Nee Tang,[2] and Patrick K. H. Lee[3,4]*

[1]School of Energy and Environment, City University of Hong Kong, Hong Kong SAR, China
[2]Facility Management and Environmental Engineering, TAL Group, Hong Kong SAR, China
[3]School of Energy and Environment and State Key Laboratory of Marine Environmental Health, City University of Hong Kong, Kowloon, Hong Kong SAR, China
[4]Climate Impact and Environmental Resilience Research Center, City University of Hong Kong, Hong Kong SAR, China

Corresponding author: Patrick K. H. Lee; B5423, Yeung Kin Man Academic Building, School of Energy and Environment, City University of Hong Kong, Tat Chee Avenue, Kowloon, Hong Kong SAR, China; E-mail: patrick.kh.lee@cityu.edu.hk; Tel: (852) 3442-4625; Fax: (852) 3442-0688.

This PDF file includes:

Supplementary Text, References, and Figures S1 to S26.

Text S1

Detailed Materials and Methods

Modeling COD removal kinetics in AS systems

Because the WWTPs were primarily designed for COD removal, with most removal occurring in the AS system, COD removal kinetics were evaluated using first-order [1] and Grau second-order [2] models. Both models were applied to AS influent and effluent COD concentration from each plant, and the best-fitting model was selected based on regression performance. The first-order model is defined as follows:

$$-\frac{dS}{dt} = \frac{QS_i}{V} - \frac{QS_e}{V} - K_1 S_e$$

where S_i and S_e are the influent and effluent COD concentrations, respectively (mg/L), Q is the influent flow rate (m^3/d), V is the tank volume (m^3), and K_1 is the first-order rate constant (d^{-1}). Under pseudo-steady-state conditions (i.e., $dS/dt \approx 0$), the equation simplifies to

$$K_1 S_e = \frac{Q(S_i - S_e)}{V}$$

The Grau second-order model is defined as follows:

$$-\frac{dS}{dt} = \left(\frac{S_e}{S_i}\right)^2 K_2 X_b$$

where K_2 is the second-order rate constant (d^{-1}) and X_b is the biomass concentration (mg/L). After linearization, the model becomes

$$\frac{VS_i}{Q(S_i - S_e)} = \frac{V}{Q} + \frac{S_i}{K_2 X}$$

Model fits were evaluated through linear regression using the lme4 package (v1.1-32) in R, with plant included as a fixed effect. For the first-order model, the regression model was as follows:

$$\frac{Q(S_i - S_e)}{V} \sim S_e + plant$$

For the Grau second-order model, the regression model was as follows:

$$\frac{VS_i}{Q(S_i - S_e)} \sim \frac{V}{Q} + plant$$

The model with a higher R^2 was selected to estimate COD removal kinetics for each plant's AS system, using the nls.mutltstart package (v1.2.0) in R.

Quantification of temporal turnover and halving time

Temporal dynamics of viral communities within individual plants were examined using time–decay slopes [3] and halving-time estimates [4]. The time–decay slope quantifies the rate of community turnover and was calculated by fitting a linear regression to log-transformed community dissimilarity (*DS*) versus log-transformed time intervals (*T*):

$$\log_{10}(DS) = a + b \log_{10} T$$

where *T* is the time between two sampling dates, *a* is the intercept, and *b* is the turnover rate. Higher *b* values indicate more rapid temporal turnover in community composition.

Halving-time, defined as the time at which community similarity declines to 50% of its initial value, was estimated using a logarithmic decay model:

$$S = c \ln(T) + int$$

where *S* is the similarity between two sampling time points *T*, *c* is the decay rate, and *int* is the intercept. Assuming an initial similarity (S_0) of 1, halving-time was calculated as follows [5]:

$$HT = e^{\left(\frac{\frac{S_0}{2} - int}{c}\right)}$$

References

1. Borghei SM, Sharbatmaleki M, Pourrezaie P, Borghei G. Kinetics of organic removal in fixed-bed aerobic biological reactor. *Bioresour Technol.* 2008;**99**:1118-24. https://doi.org/10.1016/j.biortech.2007.02.037

2. Grau P, Dohanyos M, Chudoba J. Kinetics of multicomponent substrate removal by activated sludge. *Water Res*. 1975;**9**:637-42. https://doi.org/10.1016/0043-1354(75)90169-4
3. Nekola JC, White PS. The distance decay of similarity in biogeography and ecology. *J Biogeogr*. 1999;**26**:867-78. https://doi.org/10.1046/j.1365-2699.1999.00305.x
4. Soininen J, McDonald R, Hillebrand H. The distance decay of similarity in ecological communities. *Ecography*. 2007;**30**:3-12. https://doi.org/10.1111/j.0906-7590.2007.04817.x
5. Villarino E, Watson JR, Jönsson B, Gasol JM, Salazar G, Acinas SG, et al. Large-scale ocean connectivity and planktonic body size. *Nat Commun.* 2018;**9**:142. https://doi.org/10.1038/s41467-017-02535-8

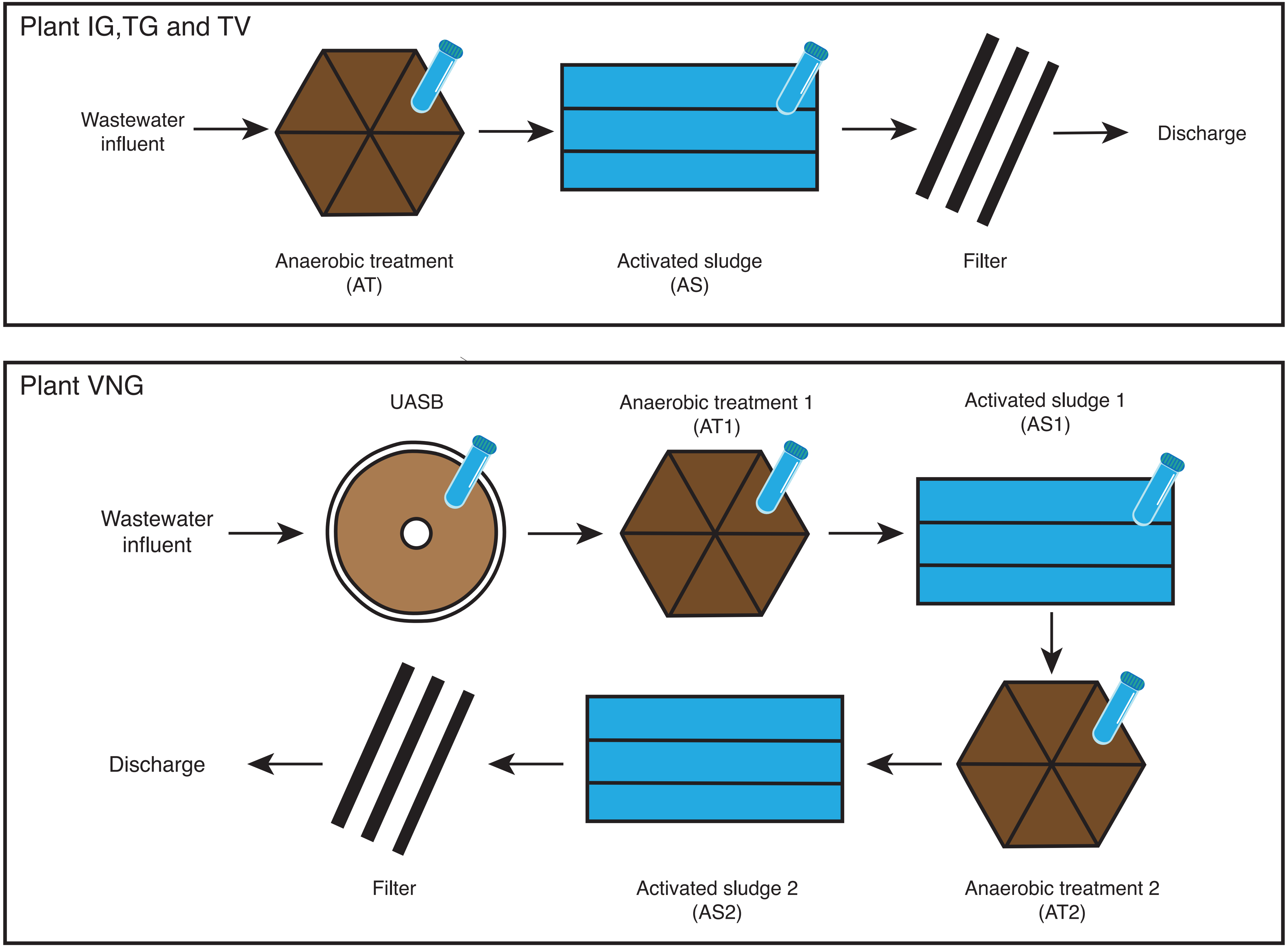

Figure S1. Schematic diagram of the wastewater treatment system setup in the four plants. Systems sampled for microbial and viral analyses are indicated.

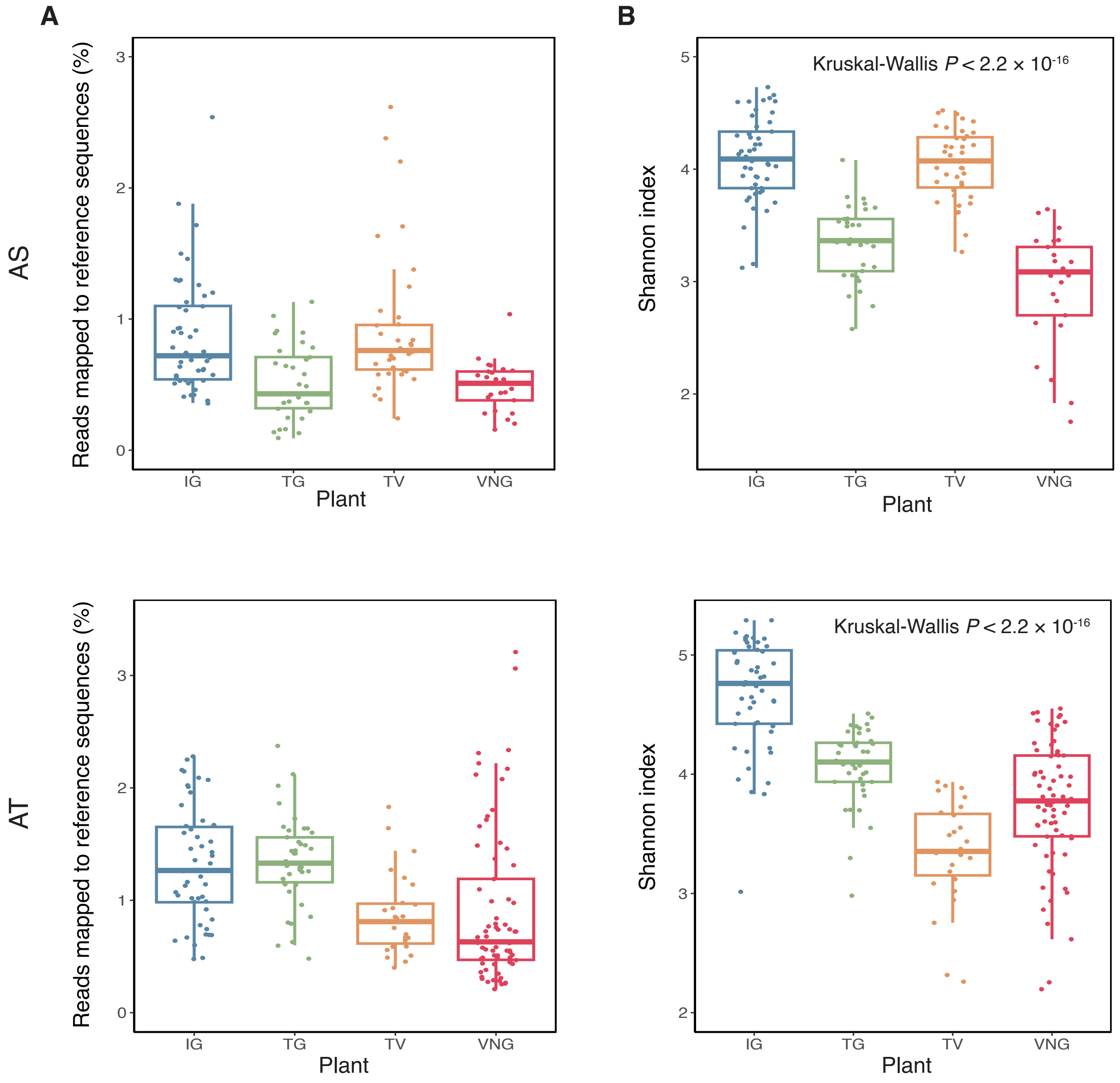

Figure S2. (A) Proportion of metagenomic reads mapped to high-quality operational taxonomic units (HQ vOTUs) and (B) Shannon diversity indices of viral communities.

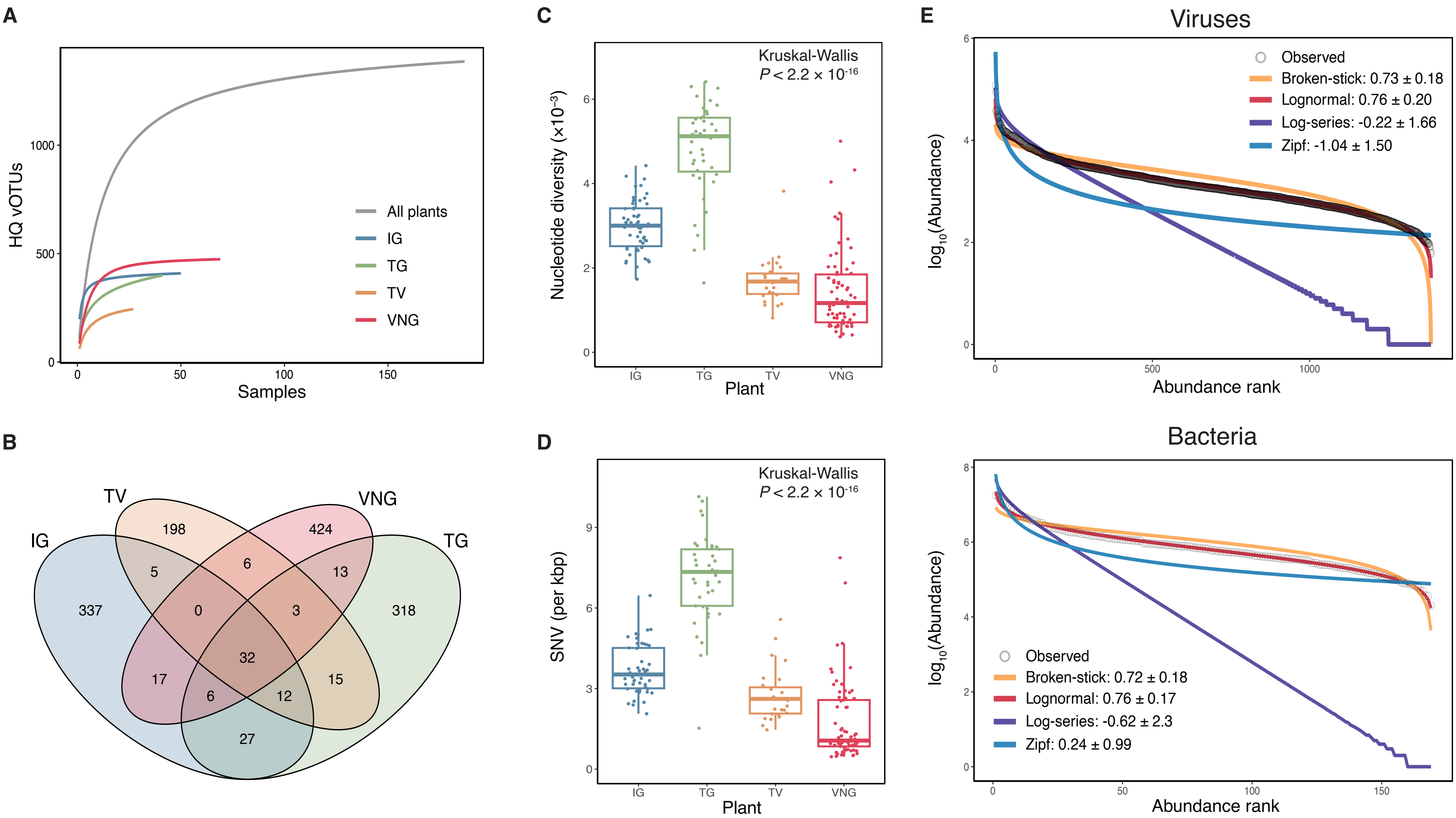

Figure S3. Distribution and diversity of high-quality viral operational taxonomic units (HQ vOTUs) in anaerobic treatment (AT) systems. (A) Accumulation curves showing HQ vOTU count as a function of sample number for individual plants and all plants combined. **(B)** Number of HQ vOTUs shared among the four plants. **(C)** Abundance-weighted nucleotide diversity of viral communities across plants. **(D)** Abundance-weighted single-nucleotide variant (SNV) density of viral communities across plants. **(E)** Abundance distributions of bacterial and viral communities fitted to four species abundance models, namely lognormal, log-series, broken-stick, and Zipf, with corresponding R^2 values indicating model fit.

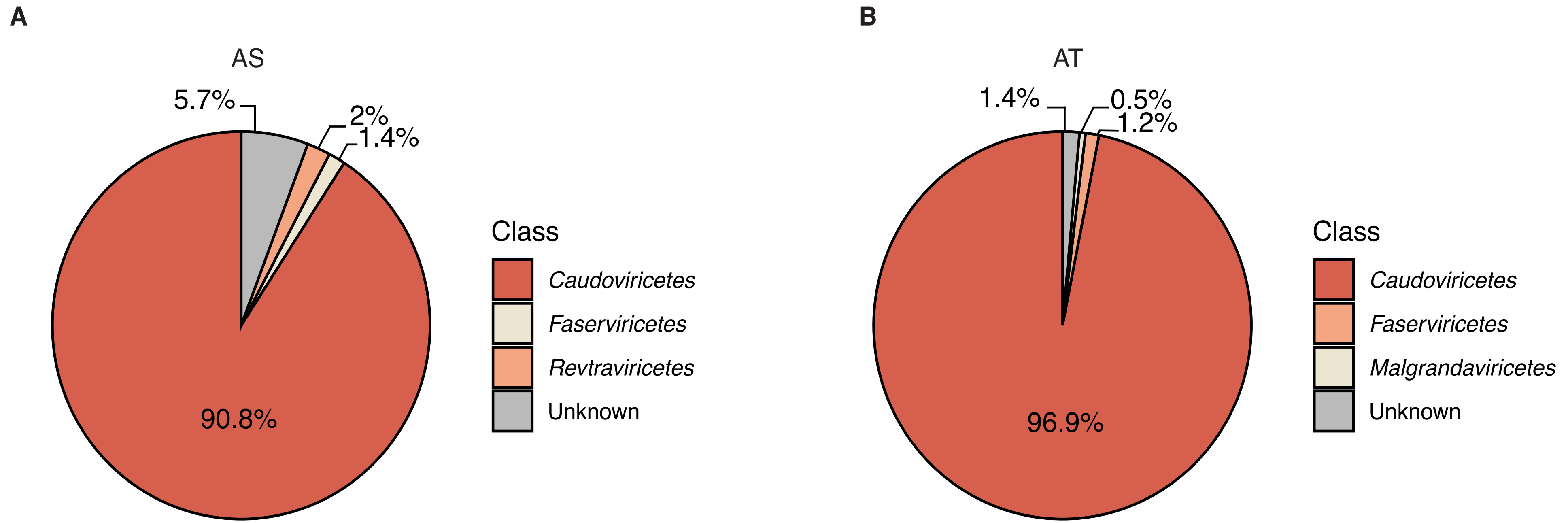

Figure S4. Taxonomic composition of viruses detected in (A) activated sludge (AS) and (B) anaerobic treatment (AT) systems at the class level.

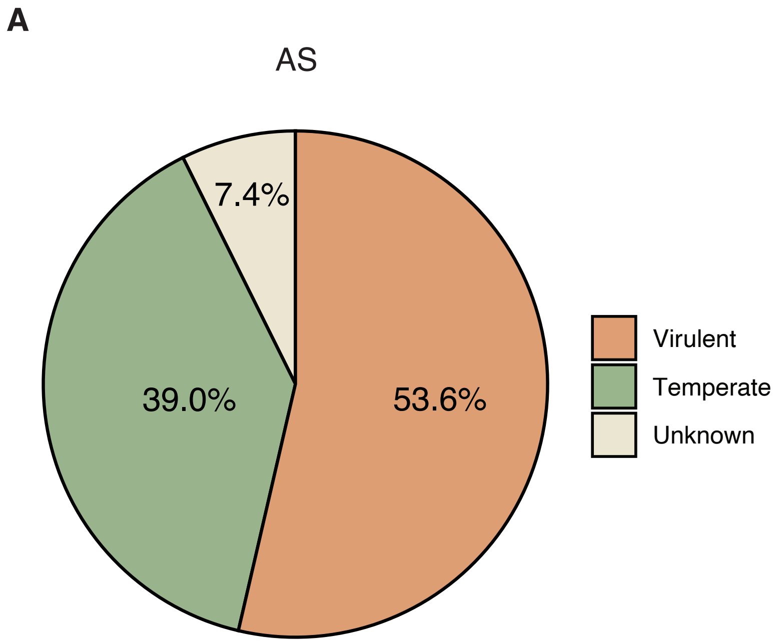

Figure S5. Predicted lifestyle of viruses detected in (A) activated sludge (AS) and (B) anaerobic treatment (AT) systems.

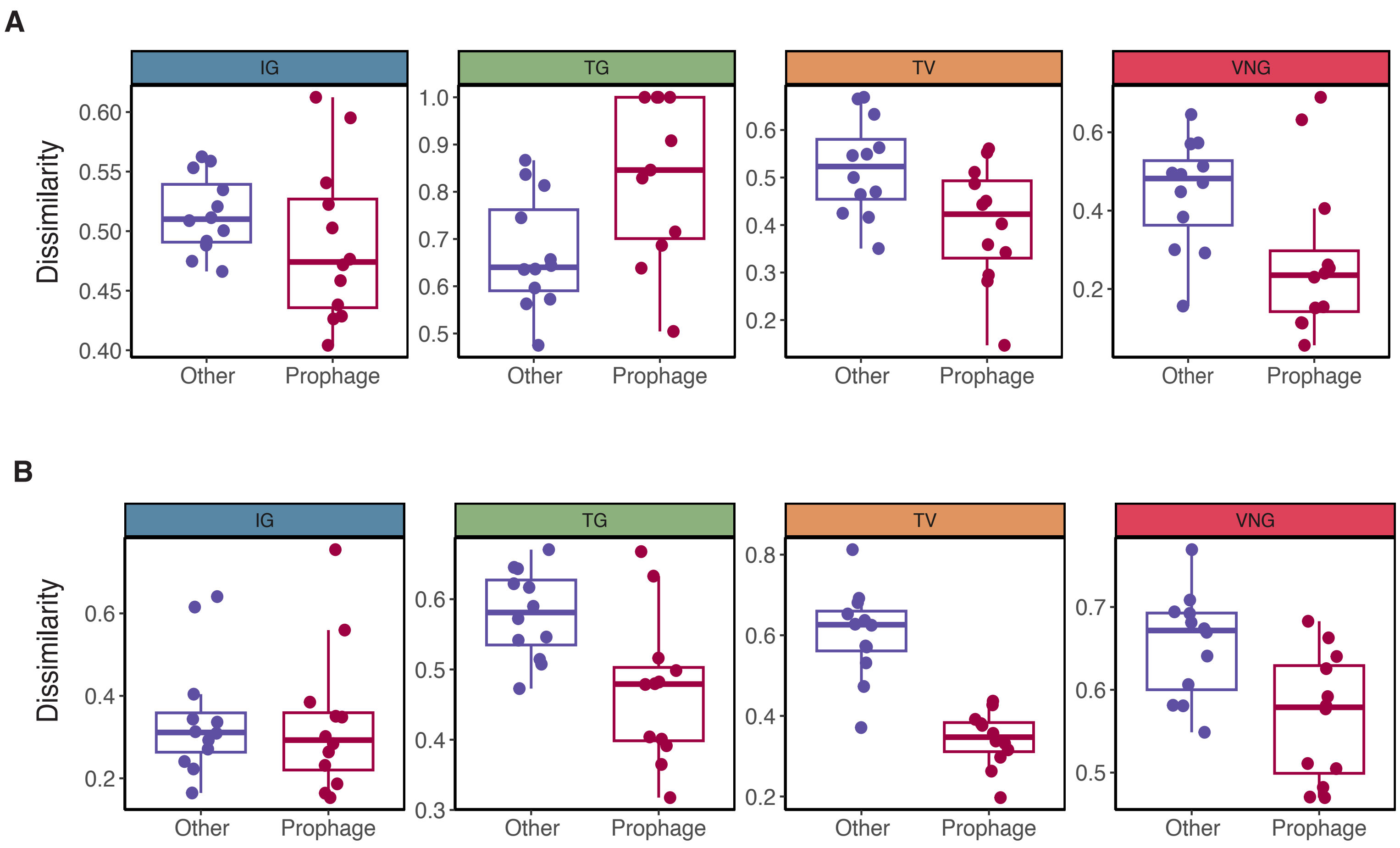

Figure S6. Consecutive-month community dissimilarly of temperate viruses in (A) activated sludge (AS) and (B) anaerobic treatment (AT) systems. Each point represents Bray–Curtis dissimilarity between temperate viral community compositions in consecutive months (month *t* vs. *t–1*) within each plant. Temperate viruses were classified as free (not identified as prophages; labeled "Others," purple) or as prophage-associated (labeled "Prophage," red).

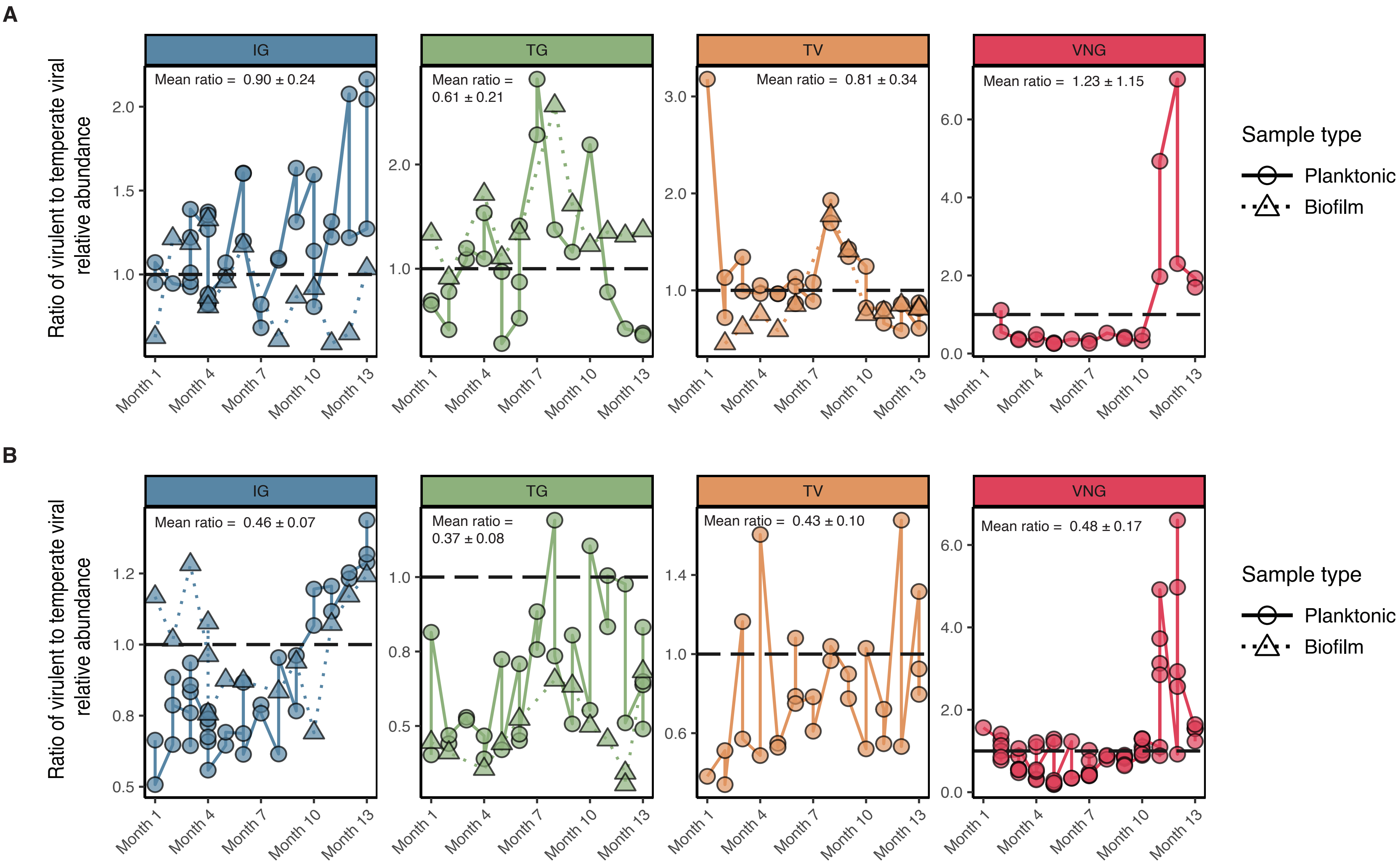

Figure S7.Temporal dynamics of the virulent-to-temperate viral relative abundance ratio in (A) activated sludge (AS) and (B) anaerobic treatment (AT) systems. Each point represents an individual sample (circles: planktonic; triangles: biofilm). Lines connect samples of the same type within each plant (solid: planktonic; dotted: biofilm). The horizontal dashed line indicates a ratio of 1, representing equal relative abundances of virulent and temperate viruses. Insets show the mean ± standard deviation of the virulent-to-temperate ratio for each plant across the sampling period. Biofilm samples were not available for plant VNG and the AT system of plant TV.

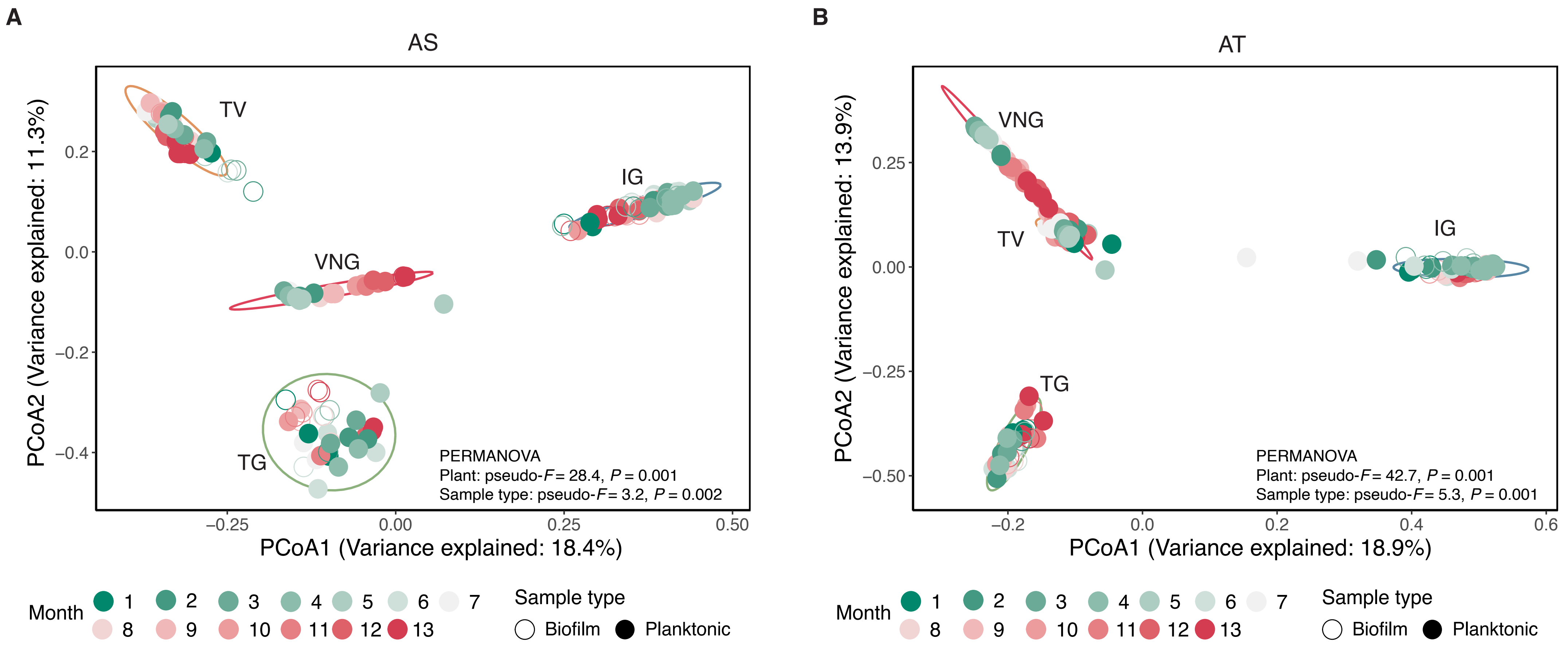

Figure S8. Principal coordinate analysis of viral community composition in (A) activated sludge (AS) and (B) anaerobic treatment (AT) systems based on Bray–Curtis dissimilarity.

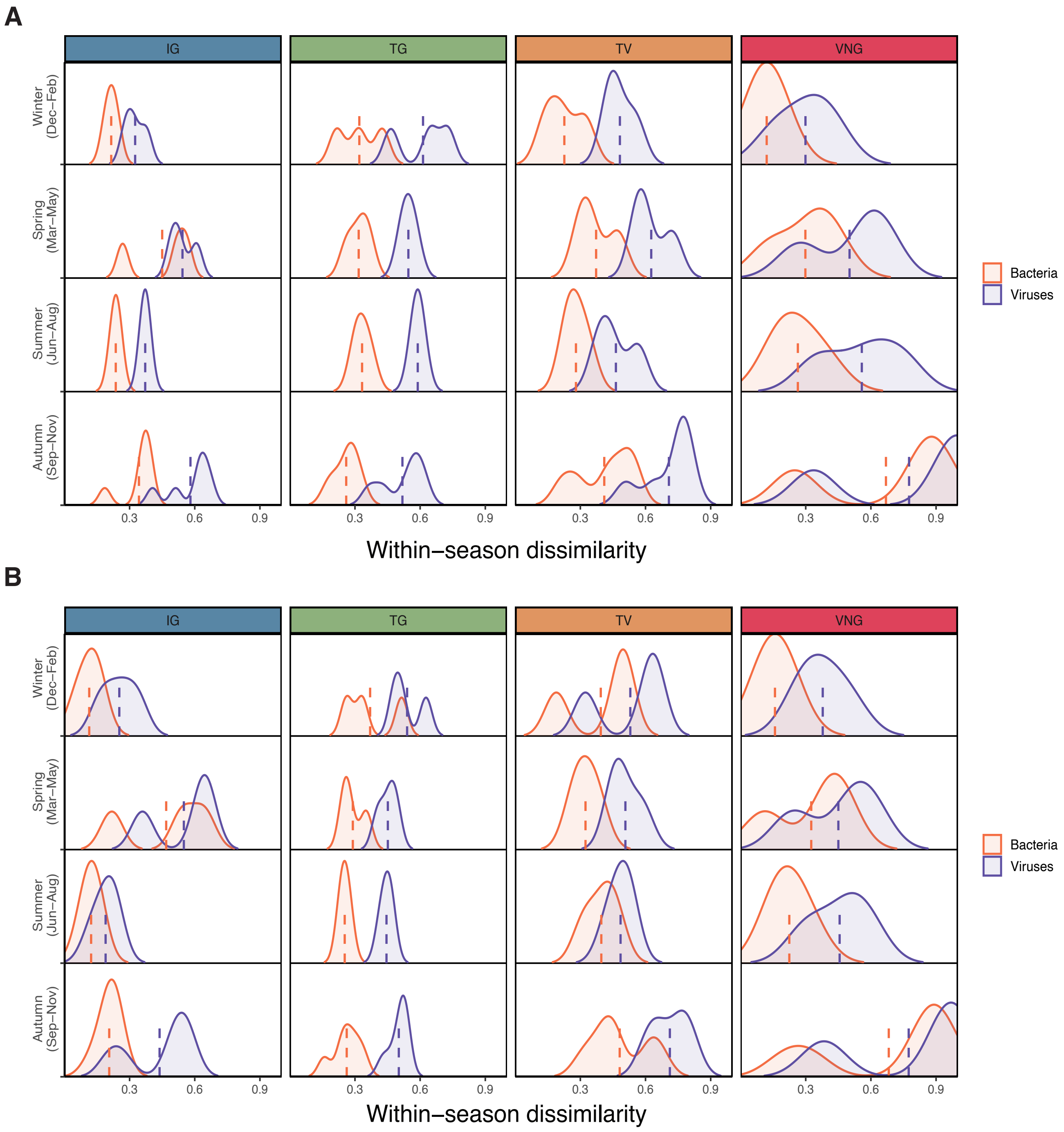

Figure S9. Seasonal patterns in viral and bacterial community dissimilarities in (A) activated sludge (AS) and (B) anaerobic treatment (AT) systems. Density plots show distributions of within-season Bray–Curtis dissimilarities for viral (purple) and bacterial (orange) communities across individual plants. Vertical dashed lines indicate the mean dissimilarity for each group.

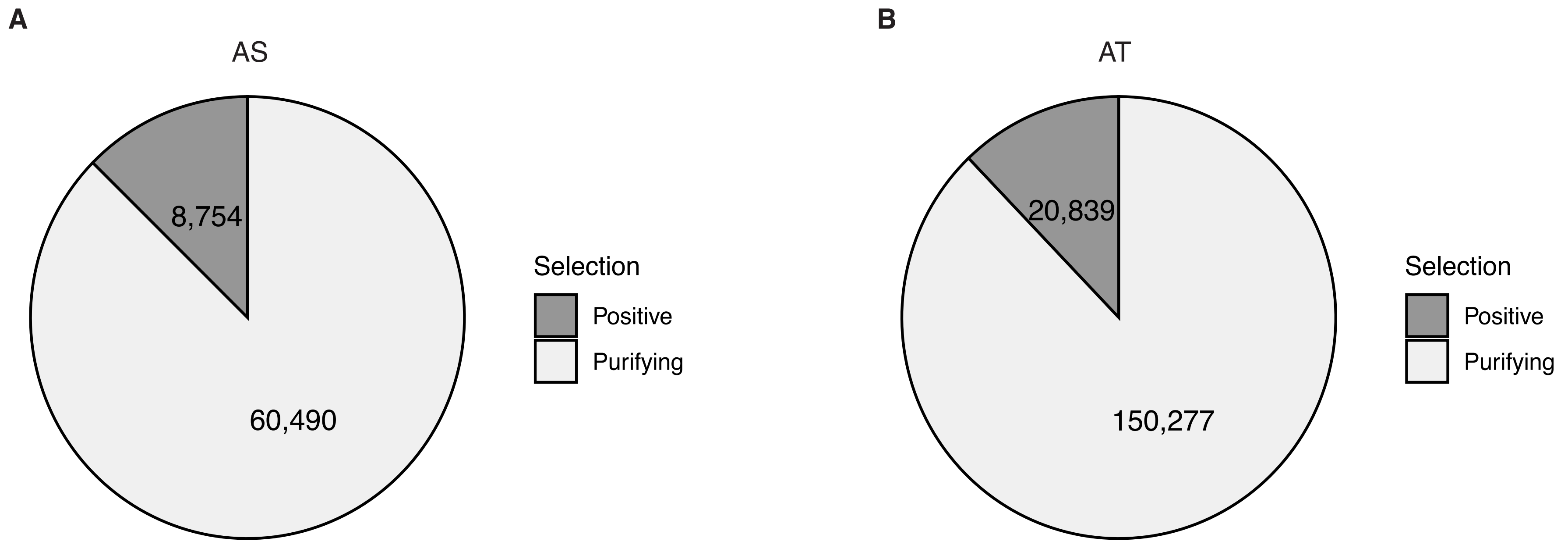

Figure S10. Proportion of viral genes under positive selection (pN/pS > 1) and purifying selection (pN/pS < 1) in (A) activated sludge (AS) and (B) anaerobic treatment (AT) systems.

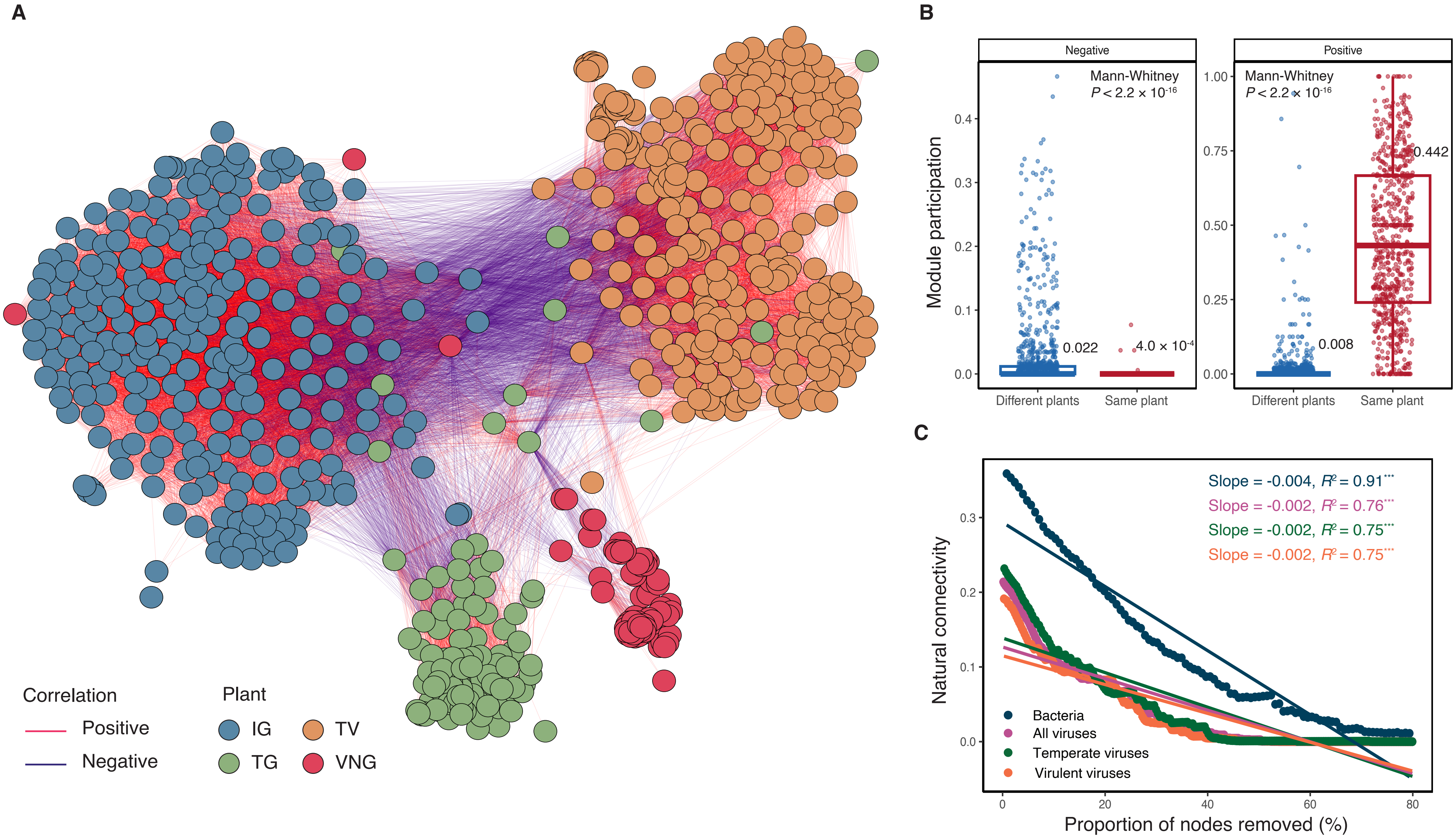

Figure S11. Co-occurrence network of viral communities in activated sludge (AS) systems. (A) Co-occurrence network of high-quality viral operational taxonomic units (HQ vOTUs). Nodes represent HQ vOTUs, and edges indicate significant co-occurrence patterns determined using a probabilistic model. Node colors correspond to the plant where each HQ vOTU exhibited the highest average relative abundance. **(B)** Module participation of HQ vOTUs within and between plants, and mean participation values are shown for each group. **(C)** Network stability analysis revealing natural connectivity as a function of the proportion of nodes removed. Slopes and R^2 values from linear regressions are shown; asterisks denote statistical significance (****P* < 0.001).

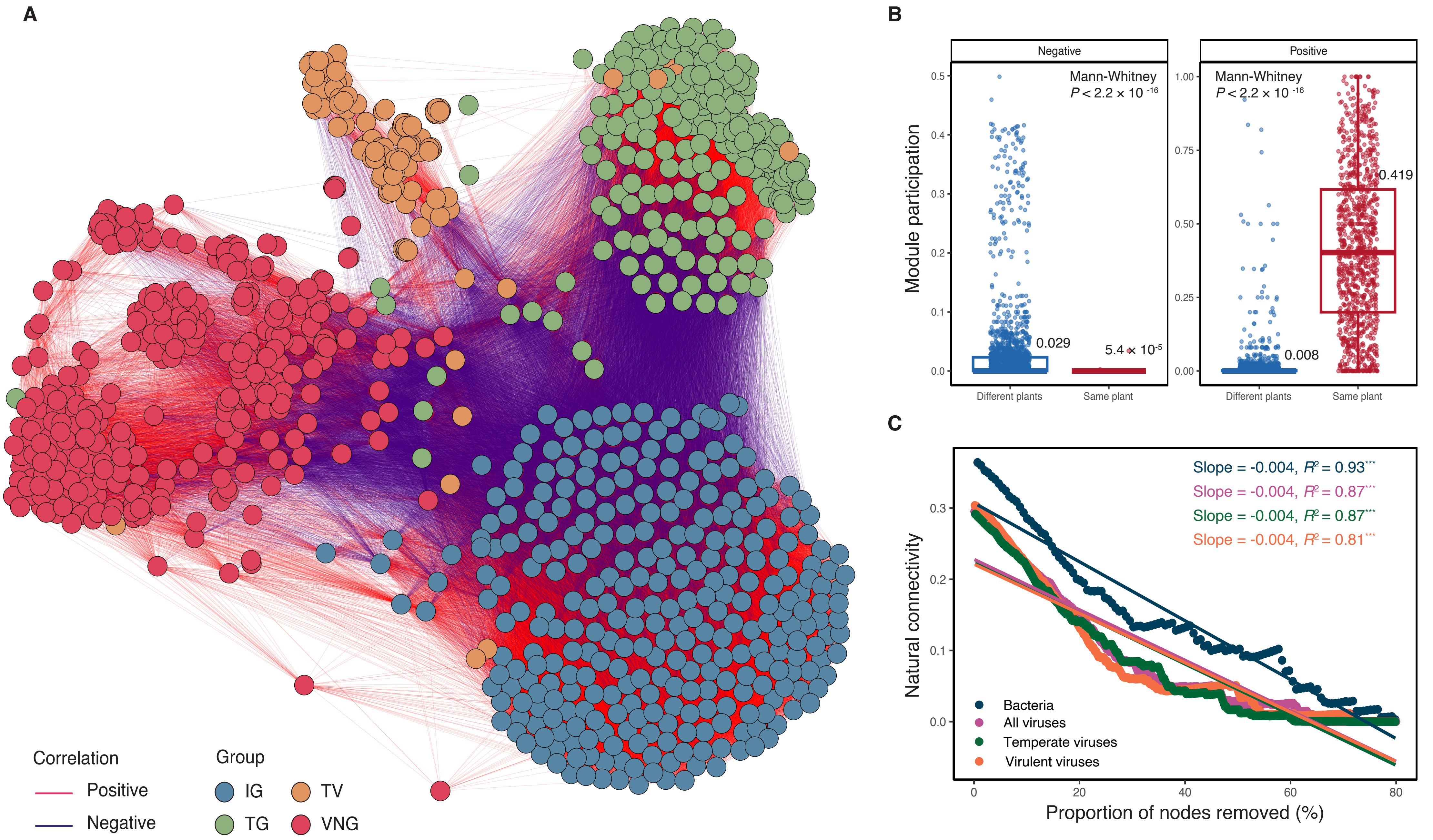

Figure S12. Co-occurrence network of viral communities in anaerobic treatment (AT) systems. (A) Co-occurrence network of high-quality viral operational taxonomic units (HQ vOTUs). Nodes represent HQ vOTUs, and edges indicate significant co-occurrence patterns determined using a probabilistic model. Node colors correspond to the plant where each HQ vOTU exhibited the highest average relative abundance. **(B)** Module participation of HQ vOTUs within and between plants, with mean participation values shown for each group. **(C)** Network stability analysis showing natural connectivity as a function of the proportion of nodes removed. Slopes and R^2 values from linear regressions are shown; asterisks indicate statistical significance (***$P < 0.001$).

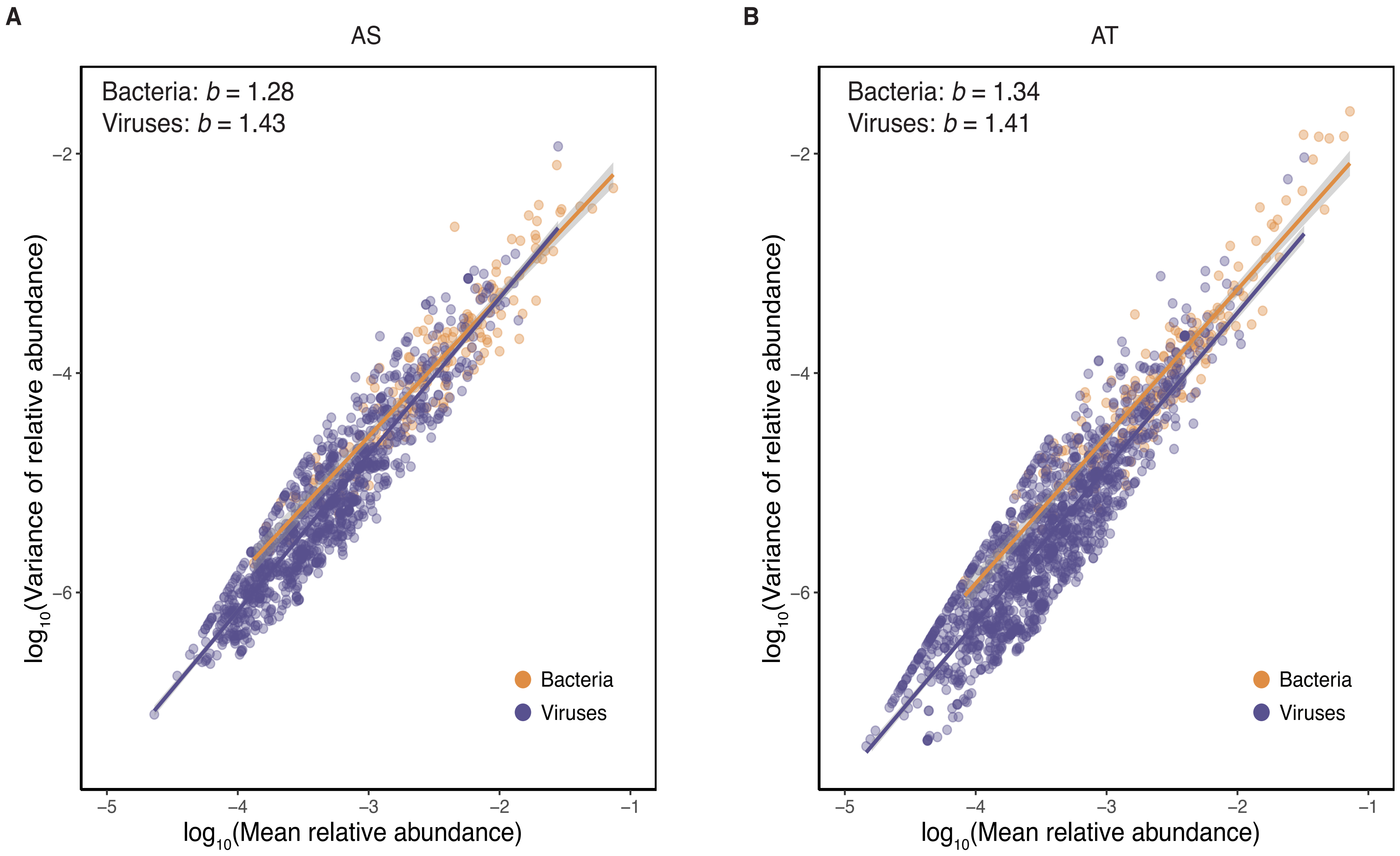

Figure S13. Taylor's power law relationships between variance and mean relative abundances of viral and bacterial communities in activated sludge (AS) and anaerobic treatment (AT) systems. Solid lines represent linear regressions fitted to log–log transformed data, and shaded areas indicate 95% confidence intervals. Estimated Taylor's law exponents (*b*-values) are shown.

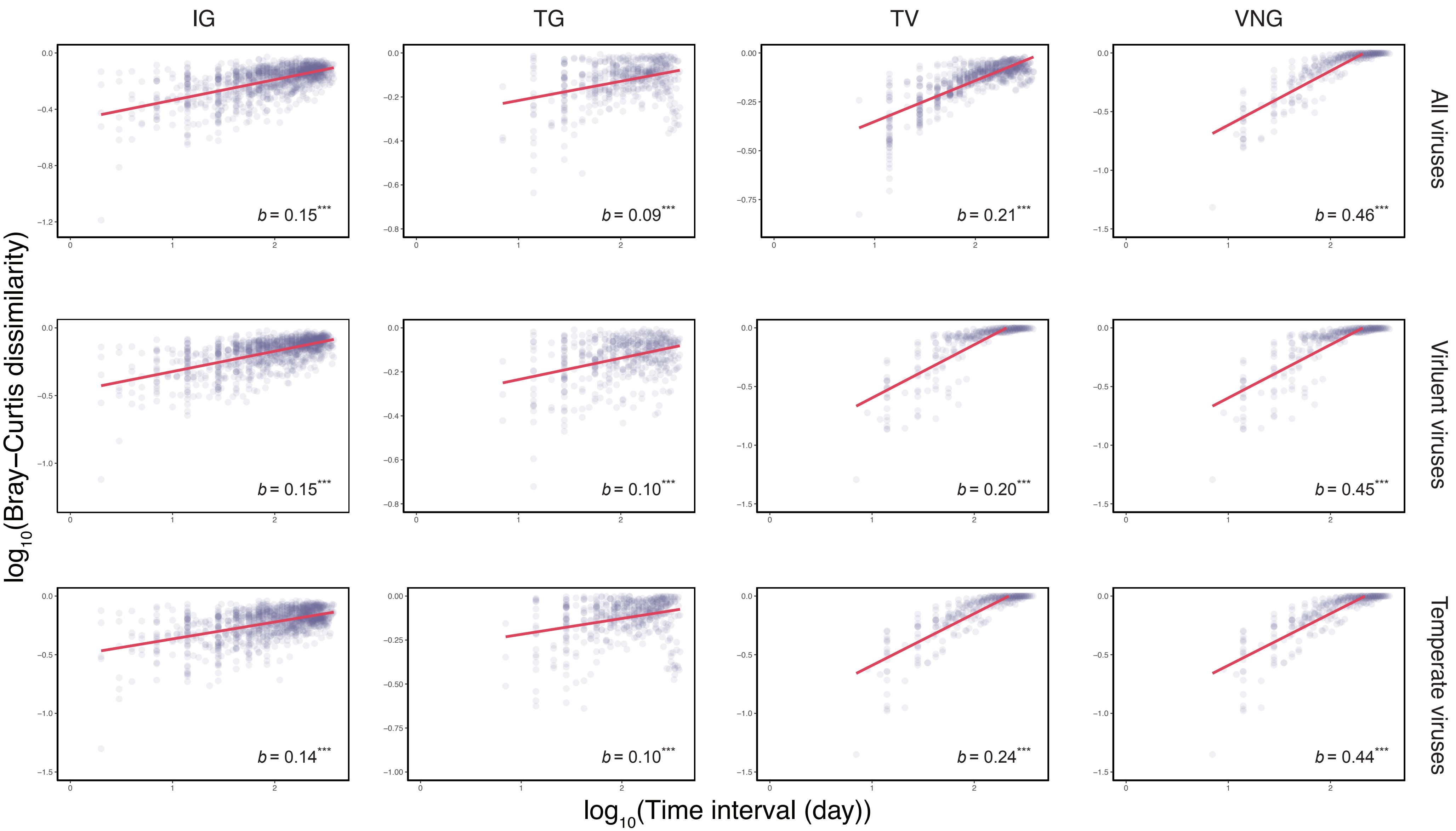

Figure S14. Time–decay relationship between viral community dissimilarity and time intervals in activated sludge (AS) systems. The red line represents a linear regression showing the decline in viral community similarity over time. The slope (*b*) indicates the rate of community turnover. Asterisks denote statistical significance: $^{**}P < 0.01$, $^{***}P < 0.001$.

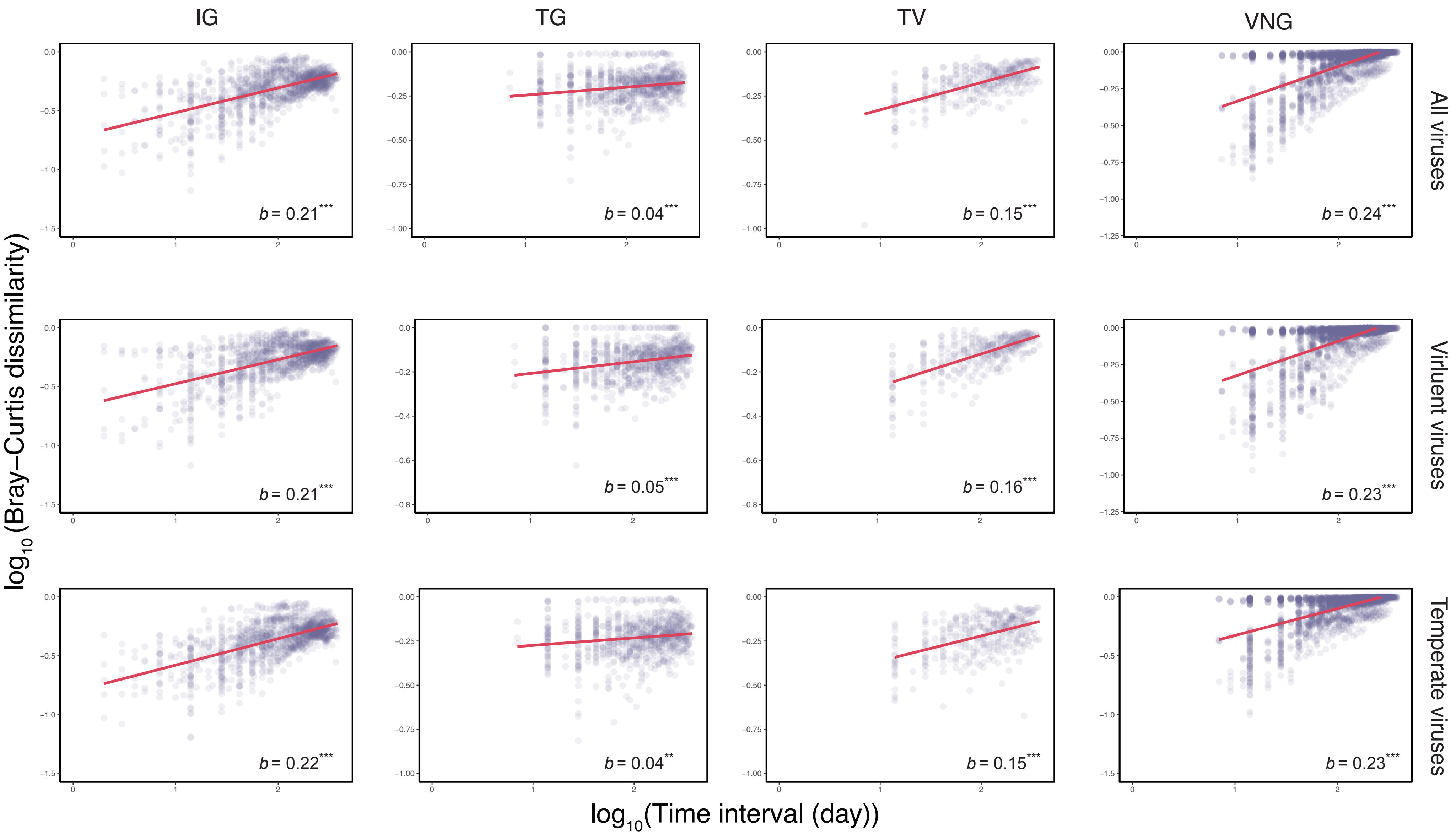

Figure S15. Time–decay relationship between viral community dissimilarity and time intervals in anaerobic treatment (AT) systems. The red line represents a linear regression showing the decline in viral community similarity over time. The slope (*b*) indicates the rate of community turnover. For plant VNG, data from three AT tanks were pooled to obtain a plant-level estimate. Asterisks denote statistical significance: $^{**}P < 0.01$, $^{***}P < 0.001$.

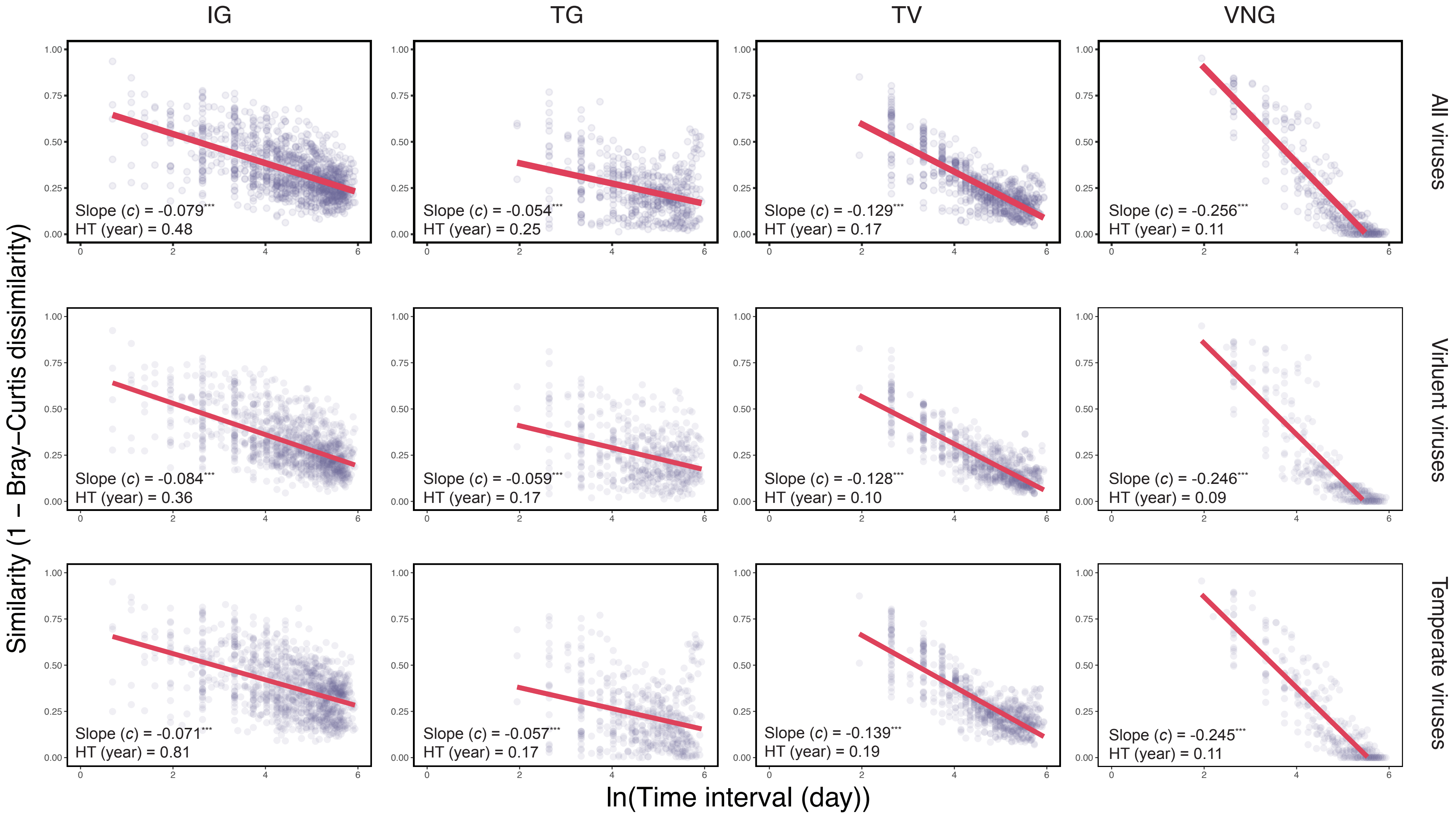

Figure S16. Temporal turnover of viral communities in activated sludge (AS) systems. Halving time (HT) of viral communities, estimated from the decay of community similarity over time. Solid lines represent fitted logarithmic decay models, and the turnover rate (slope *c*) indicates the rate of similarity loss. HT denotes the time required for community similarity to decrease by half. Asterisks denote statistical significance: ****P* < 0.001, ***P* < 0.01, **P* < 0.05.

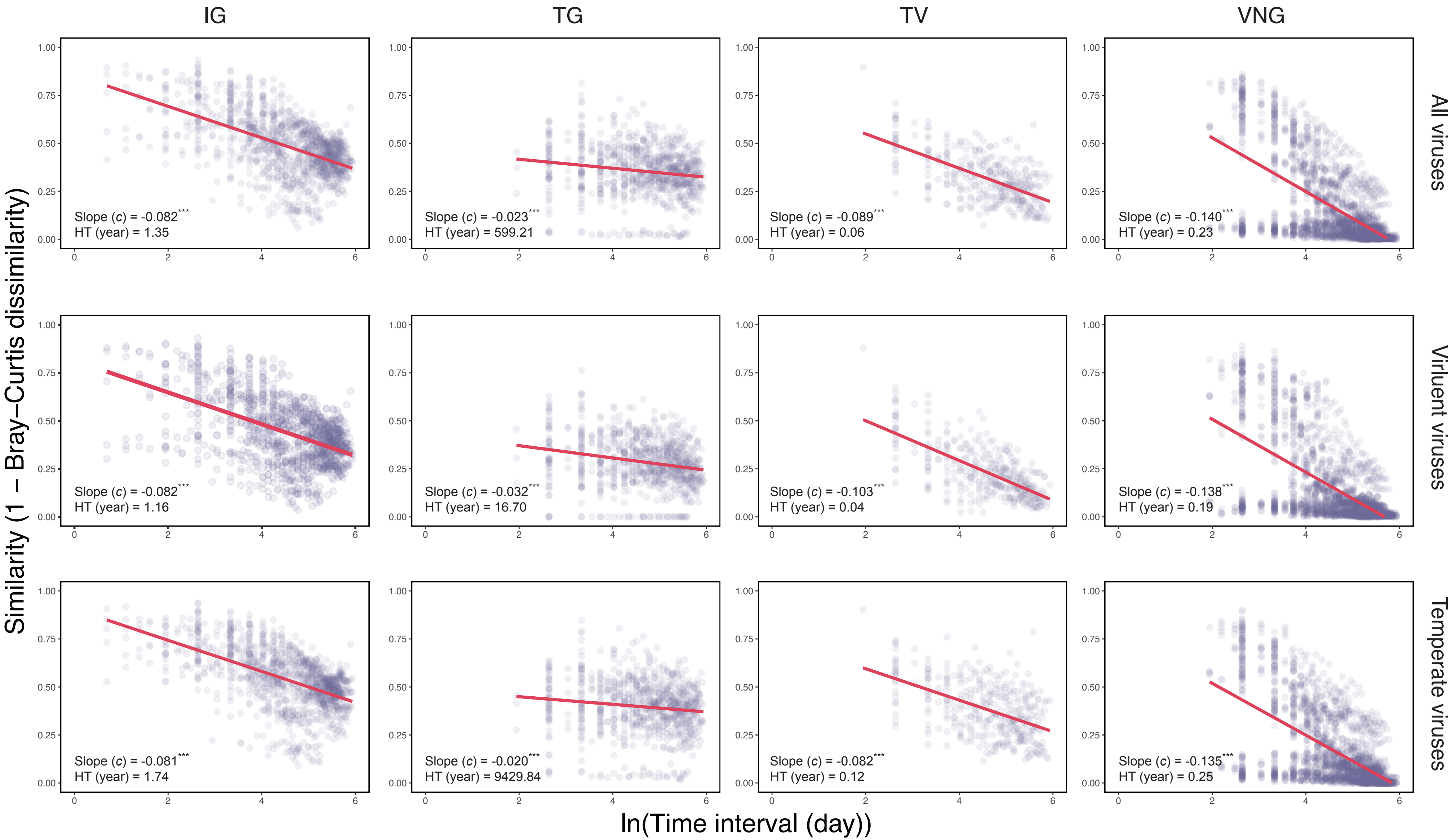

Figure S17. Temporal turnover of viral communities in anaerobic treatment (AT) systems. Halving time (HT) of viral communities, estimated from the decay of community similarity over time. Solid lines represent fitted logarithmic decay models, and the turnover rate (slope *c*) indicates the rate of similarity loss. HT denotes the time required for community similarity to decrease by half. For plant VNG, data from three AT tanks were pooled to obtain a plant-level estimate. Asterisks denote statistical significance: ***$P < 0.001$, **$P < 0.01$, *$P < 0.05$.

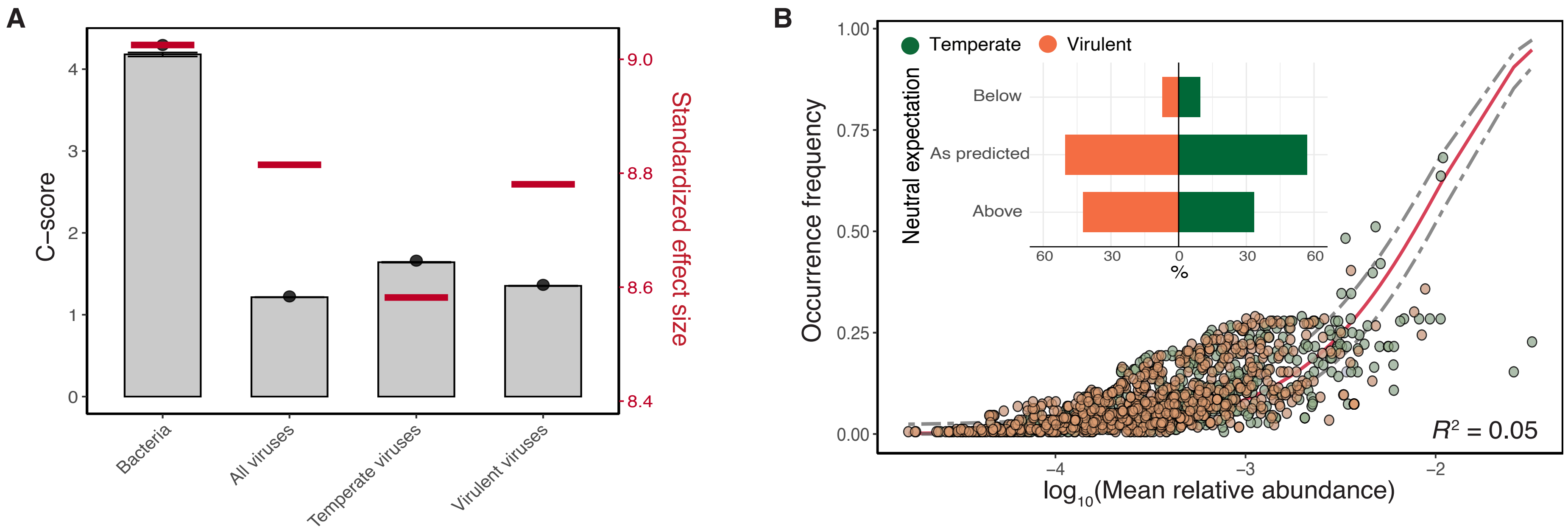

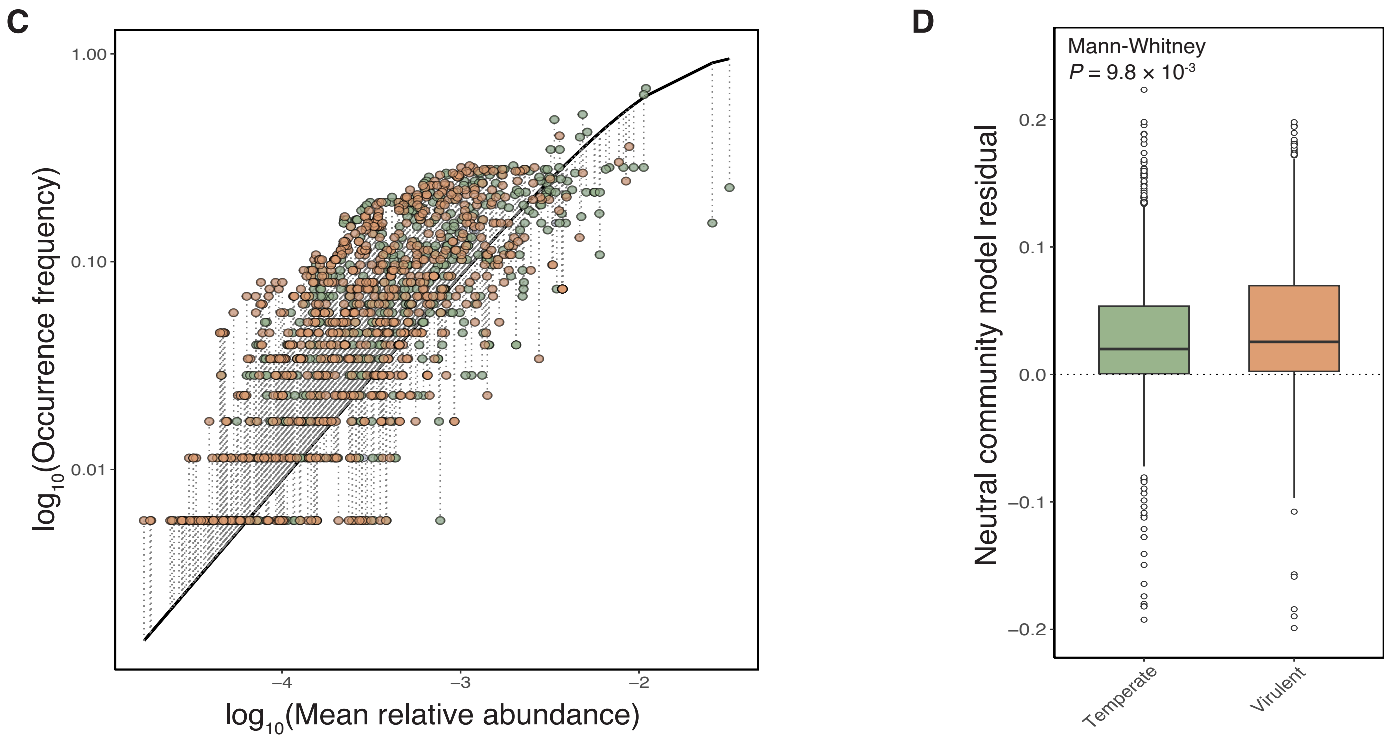

Figure S18. Ecological processes shaping viral community assembly in anaerobic treatment (AT) systems. (A) C-score analysis was performed using null models for bacterial communities and all, temperate, and virulent viruses. Dots indicate observed C-scores, and bars show simulated C-scores with 95% confidence intervals. Standardized effect sizes less than −2 and greater than 2 indicate significant aggregation and segregation, respectively. **(B)** Fit of the Sloan neutral model to viral communities. Each point represents a HQ vOTU, colored by predicted lifestyle (temperate or virulent). The solid line shows the neutral model prediction, and dashed lines indicate the 95% confidence interval. R^2 denotes model fit. The inset bar chart shows the number of HQ vOTUs falling below, within, or above neutral expectations. **(C)** Residuals from the neutral model based on log-transformed HQ vOTU prevalence. **(D)** Comparison of neutral model residuals between temperate and virulent HQ vOTUs.

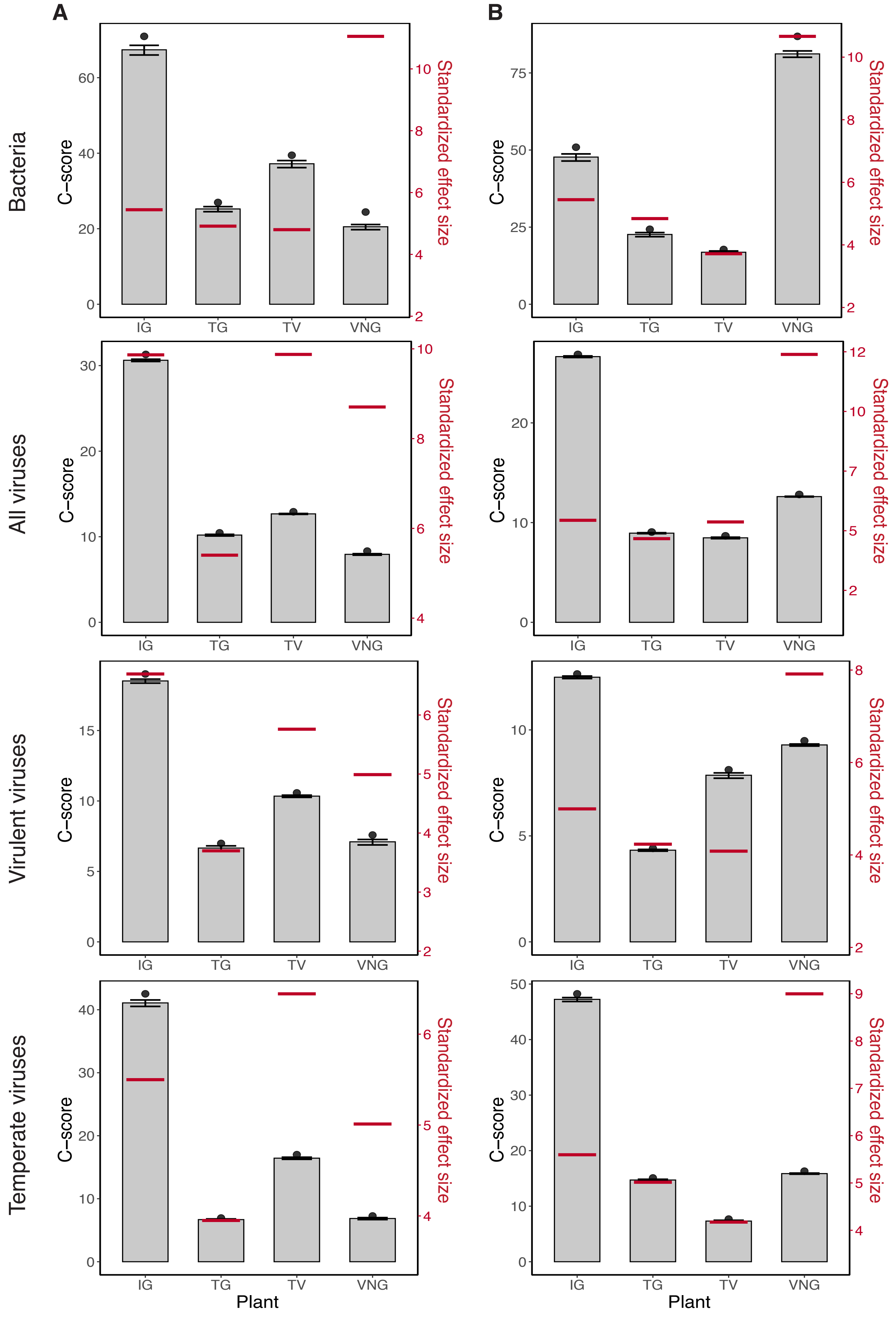

Figure S19. Temporal dynamics of C-score metrics in microbial communities from (A) activated sludge (AS) and (B) anaerobic treatment (AT) systems. Observed C-scores (points) and null model–simulated C-scores with 95% confidence intervals (bars) are shown for individual plants over time. Standardized effect sizes less than −2 and greater than 2 indicate significant aggregation or segregation, respectively. For plant VNG, data from three AT tanks were pooled to obtain a plant-level estimate.

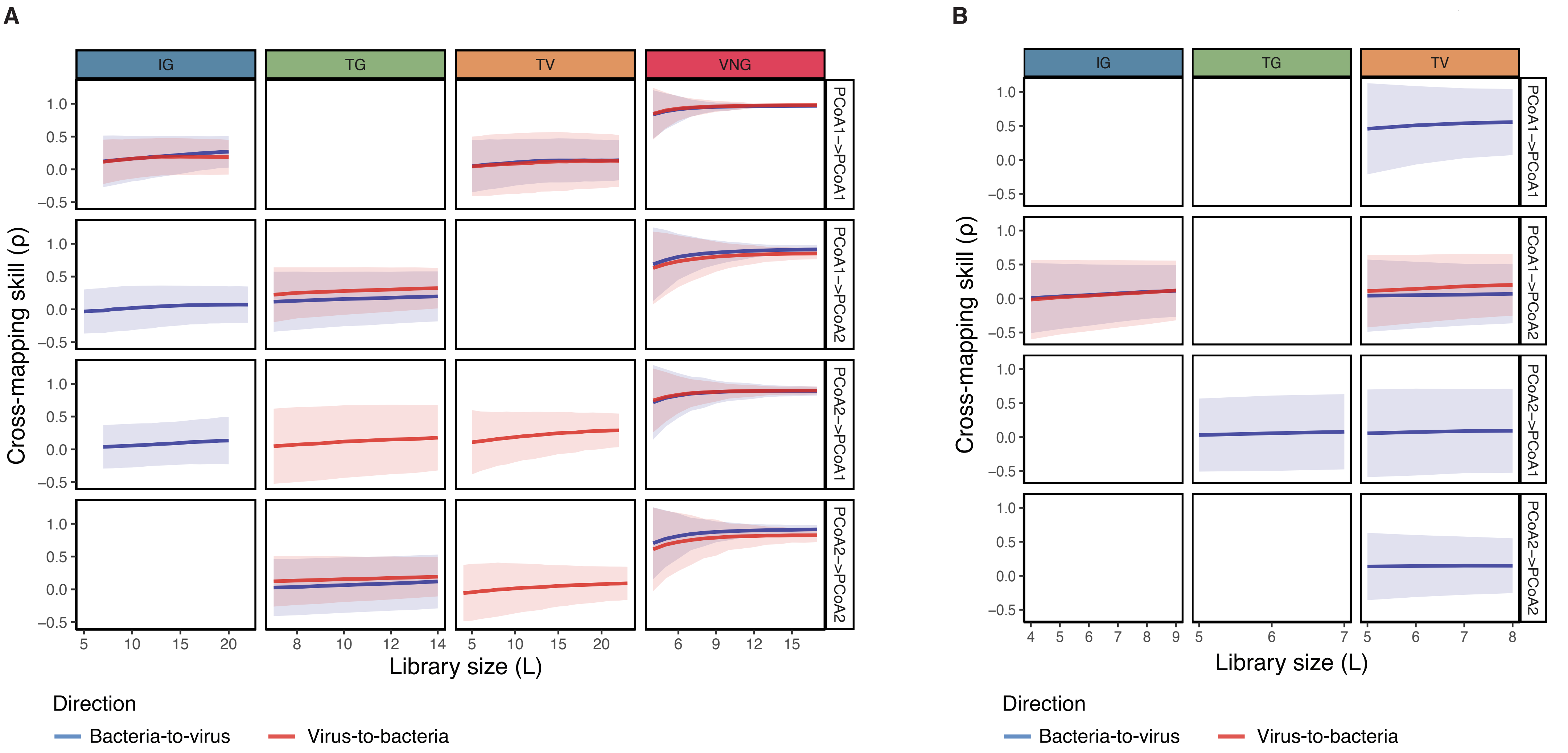

Figure S20. Directional causality between viral and bacterial community dynamics within individual WWTPs over time, shown for (A) planktonic and (B) biofilm communities. Directional causality was assessed using convergent cross mapping (CCM). Viral and bacterial community dynamics were represented by their first two principal coordinate axes (PCoA1 and PCoA2), derived from Bray–Curtis dissimilarities. Each panel displays CCM convergence curves for a given plant and PCoA axis pair (PCoAi to PCoAj), with cross-map skill (ρ) plotted against library size (L). Red lines denote the viral-to-bacterial direction; blue lines denote the bacterial-to-viral direction. Shaded areas represent 95% confidence intervals from bootstrap iterations. Only curves meeting positive CCM convergence criteria (i.e., increasing ρ with L and positive ρ at the maximum library size) are shown. Biofilm samples were not available for plant VNG.

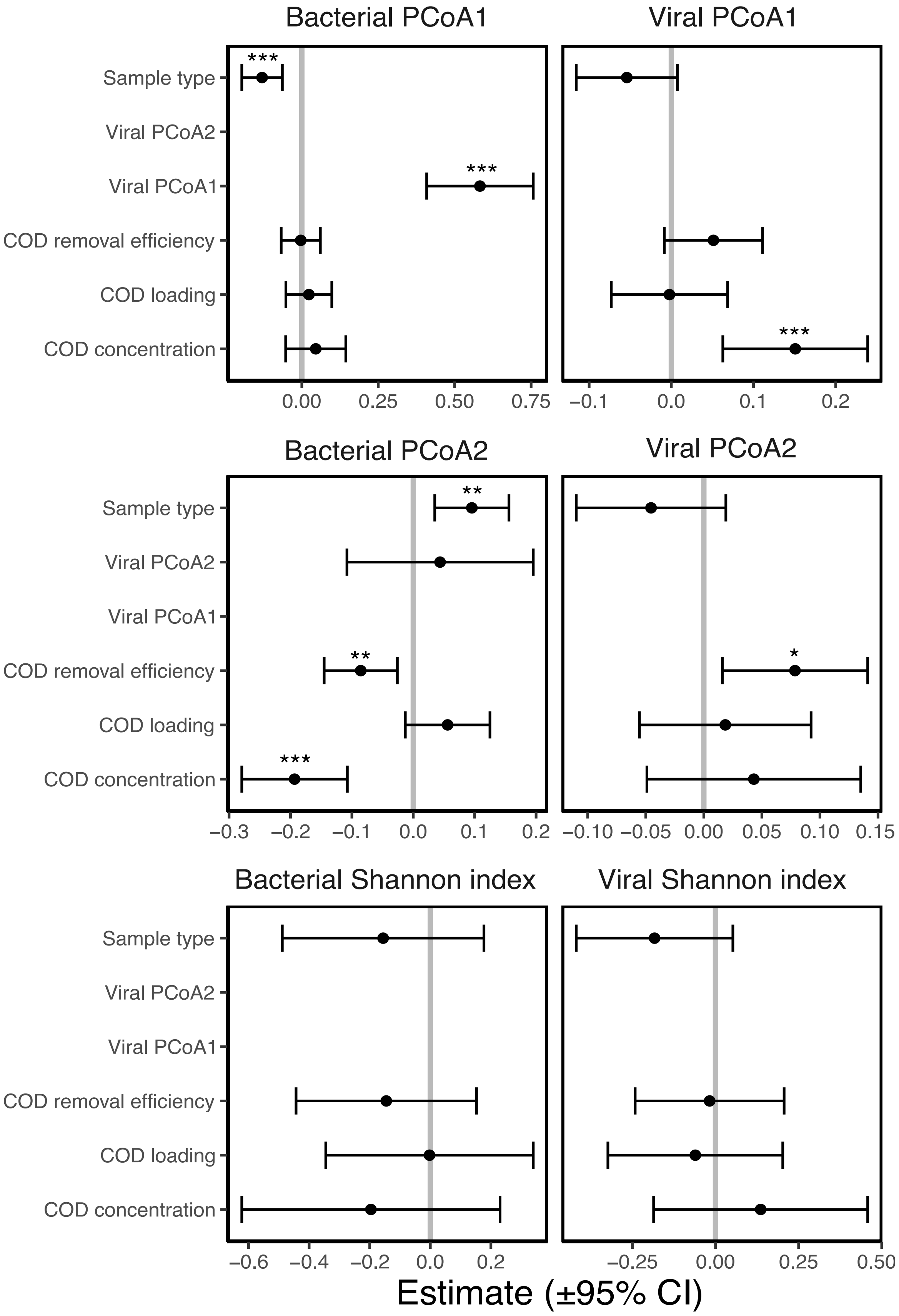

Figure S21. Model-averaged parameter estimates from optimal generalized linear mixed models exhibiting associations between COD-related parameters and microbial community metrics in activated sludge (AS) systems. The Shannon index reflects α-diversity, whereas PCoA1 and PCoA2 represent β-diversity. Mean estimates with 95% credible intervals are shown. Asterisks denote statistical significance: ***P < 0.001, **P < 0.01, *P < 0.05.

A

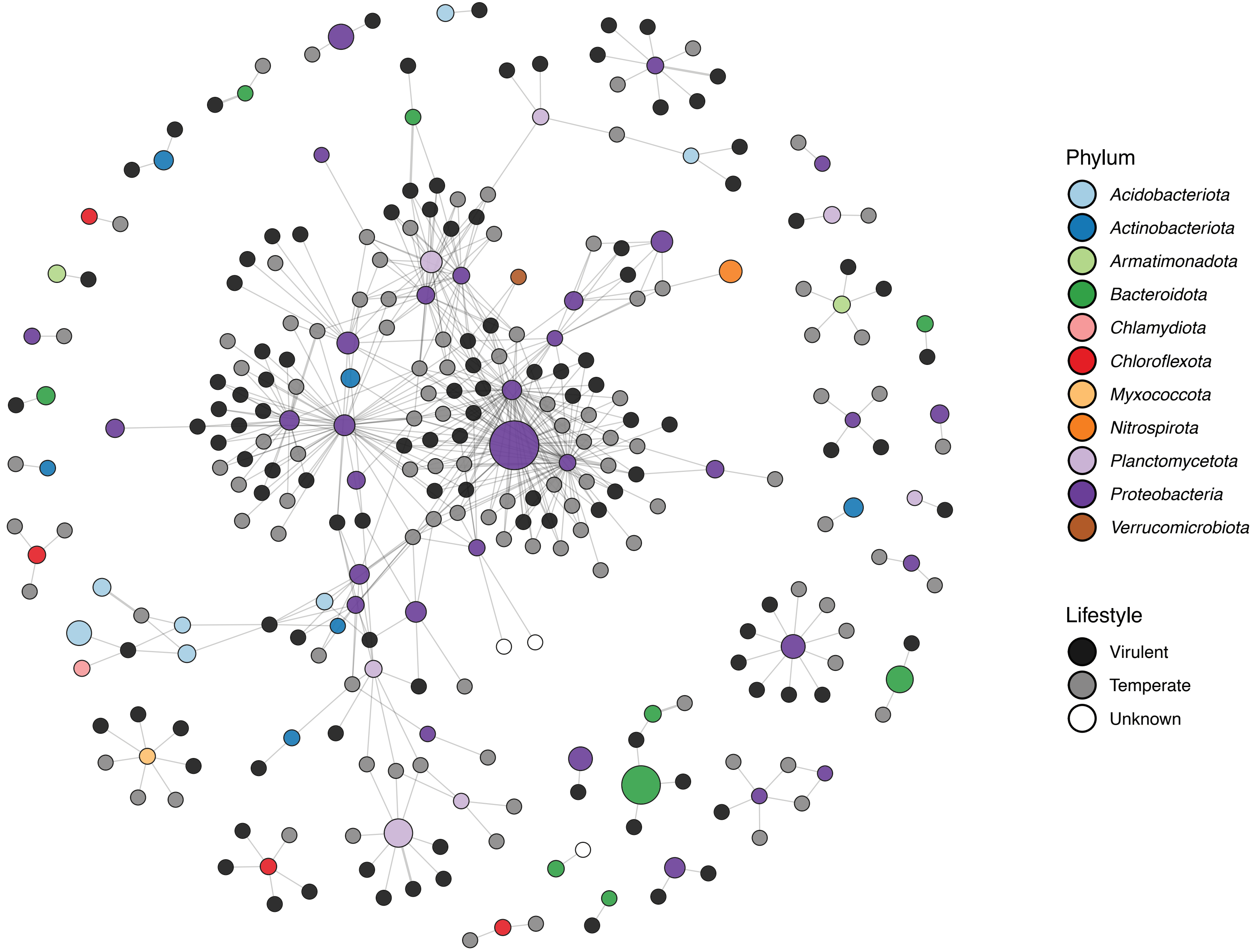

Phylum
Acidobacteriota
Actinobacteriota
Armatimonadota
Bacteroidota
Chlamydiota
Chloroflexota
Myxococcota
Nitrospirota
Planctomycetota
Proteobacteria
Verrucomicrobiota
Lifestyle
Virulent
Temperate
Unknown

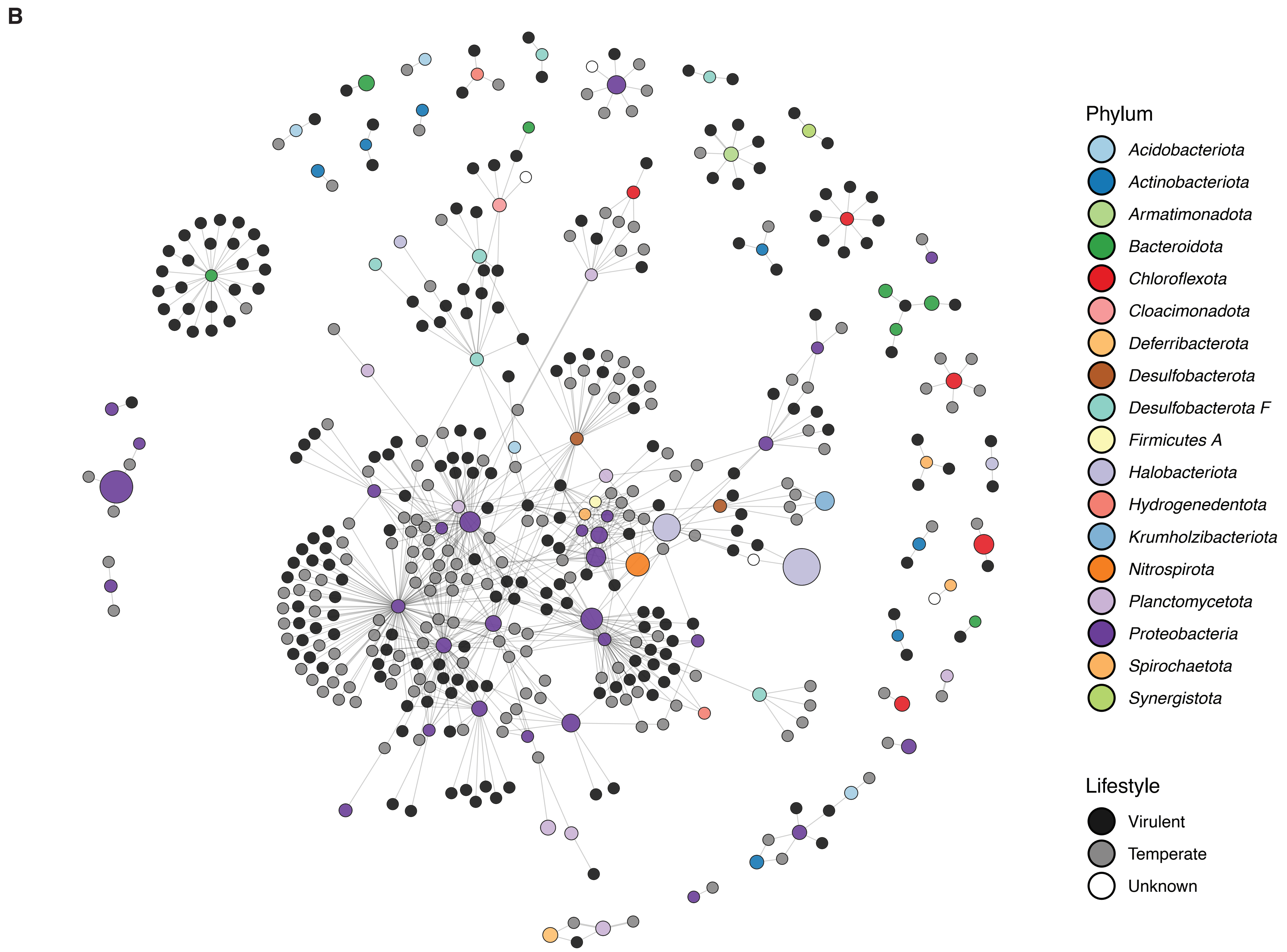

Figure S22. Predicted virus–host linkages in (A) activated sludge (AS) and (B) anaerobic treatment (AT) systems. Linkages were inferred primarily from CRISPR spacer matches and supplemented with iPHoP predictions (FDR ≤ 10%). Each node represents an individual vOTU or an individual putative bacterial host (HQ rMAG). Nodes are colored by predicted lifestyle (vOTUs) or by phylum (GTDB-Tk taxonomy; hosts), and node size is scaled by average relative abundance (hosts only).

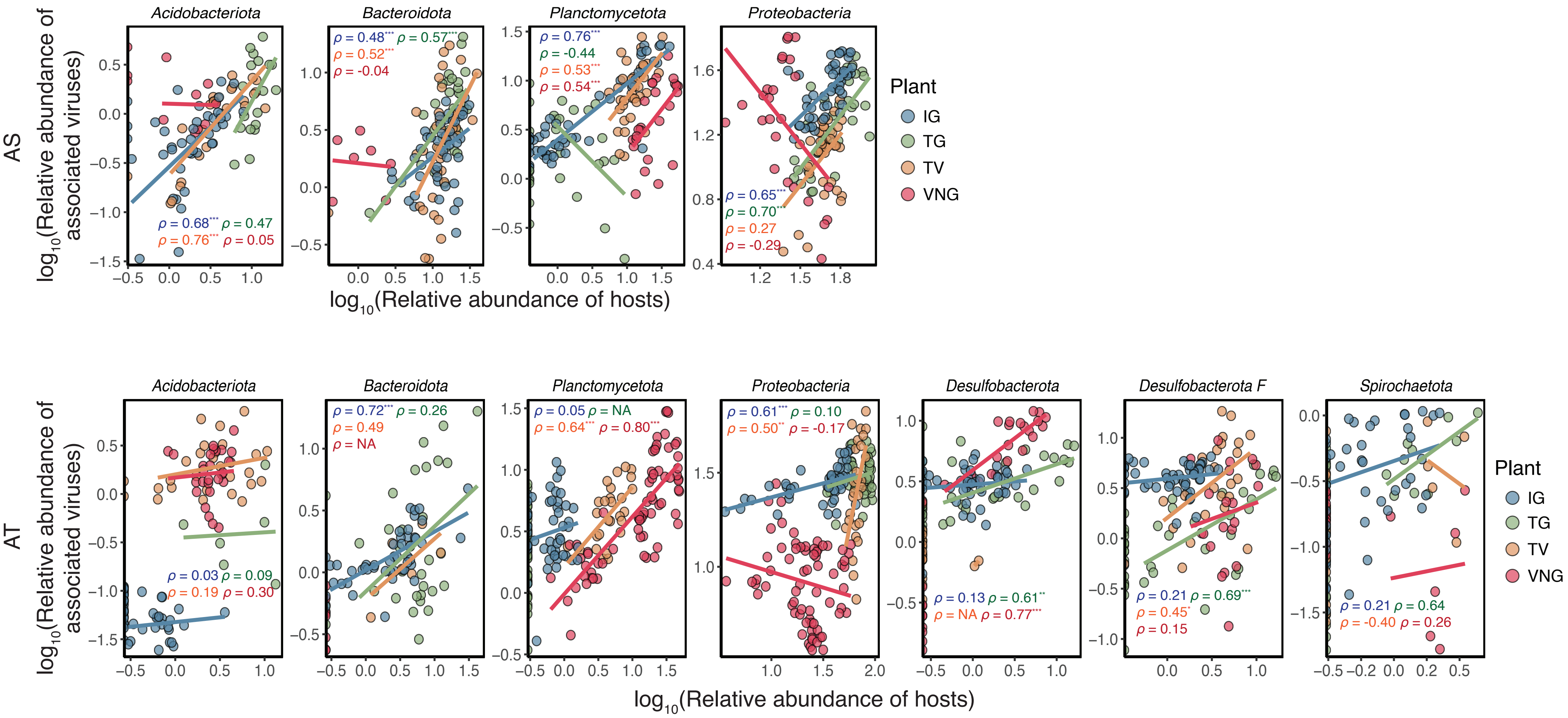

Figure S23. Relationships between the relative abundances of bacterial phyla and their associated viruses in activated sludge (AS) and anaerobic treatment (AT) systems. Only bacterial phyla detected in four plants within each system were included. Associations were assessed using Spearman's rank correlation. Asterisks denote statistical significance: ****P* < 0.001, ***P* < 0.01, **P* < 0.05. "NA" denotes that the corelations could not be computed due to limited number of data points.

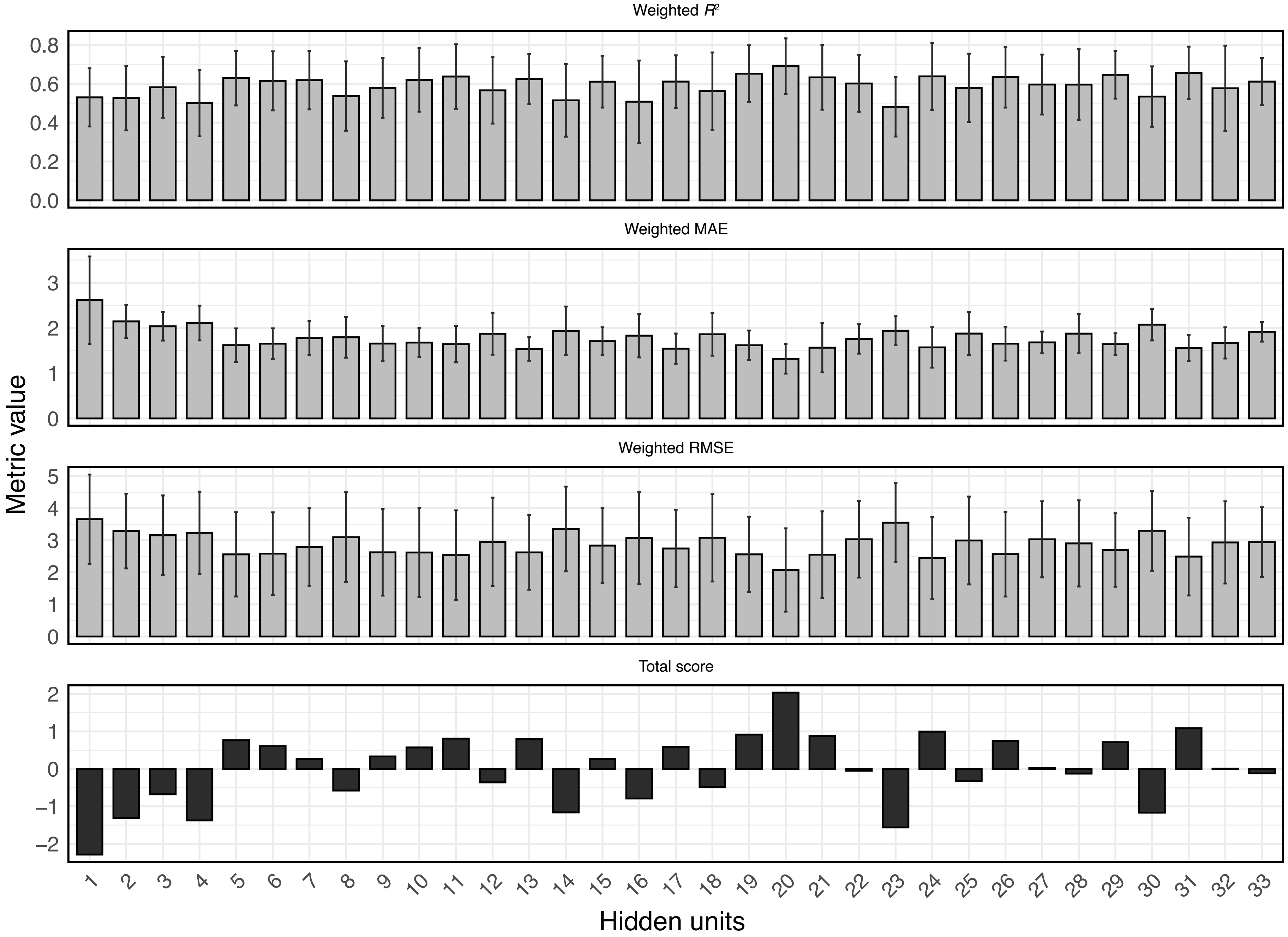

Figure S24. Prediction performance of neural networks with varying numbers of hidden units evaluated on the testing dataset. Performance metrics include weighted R^2, weighted mean absolute error (MAE), weighted root mean square error (RMSE), and total score.

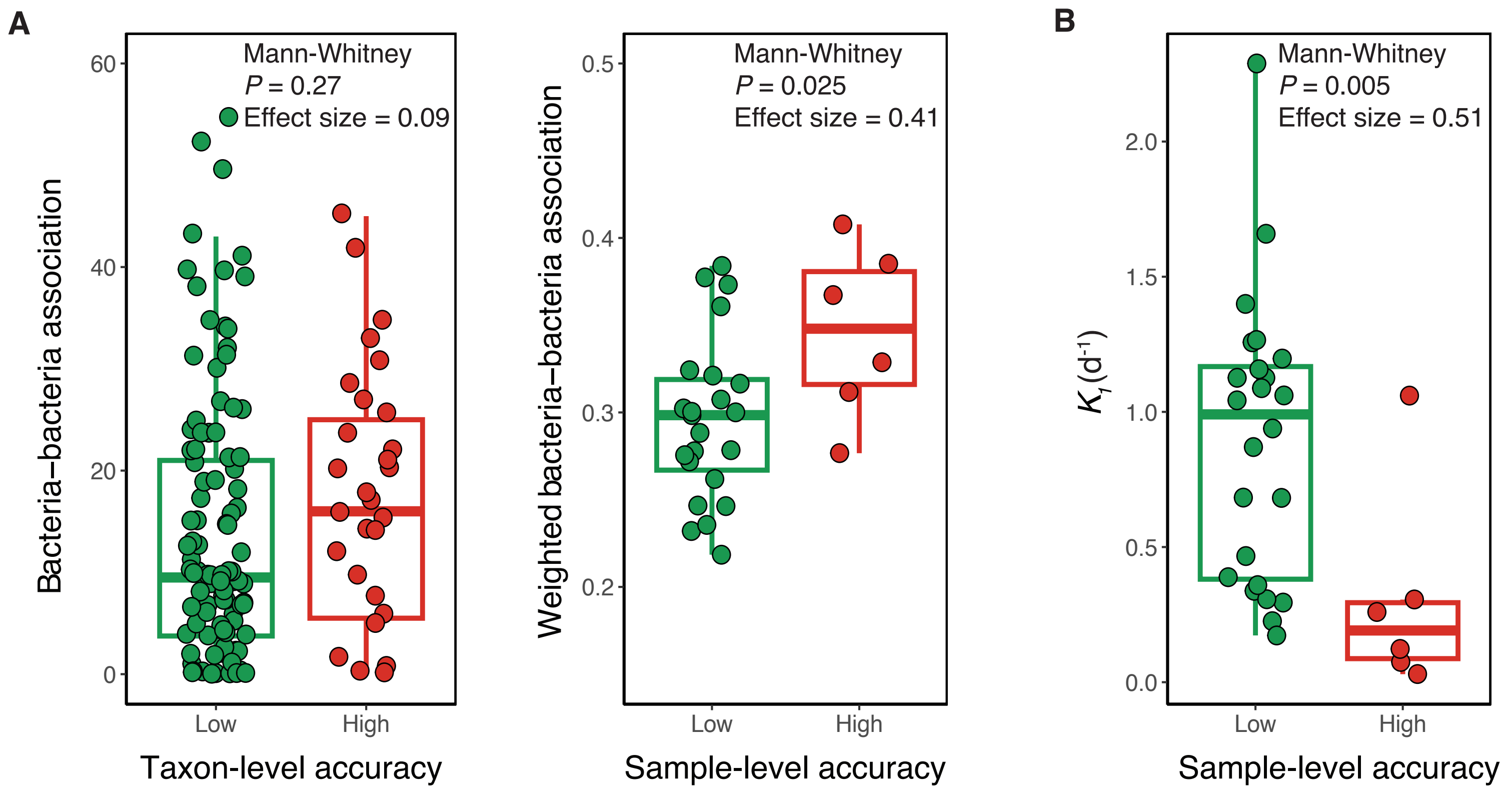

Figure S25. Bacteria–bacteria associations and first-order COD removal rate constants in samples classified as high or low accuracy. (A) Taxon-level co-occurrence associations were calculated by summing pairwise associations between each taxon and all other bacterial taxa. Sample-level association scores were computed by weighting pairwise association strengths by the relative abundances of interacting taxa and summing across all taxon pairs. Statistical comparisons were performed using the Mann–Whitney test, with corresponding effect sizes reported. **(B)** First-order COD removal rate constants (K_1) were estimated for each sample and compared between high and low accuracy groups.Statistical comparisons were performed using the Mann–Whitney test, with the corresponding effect size reported.

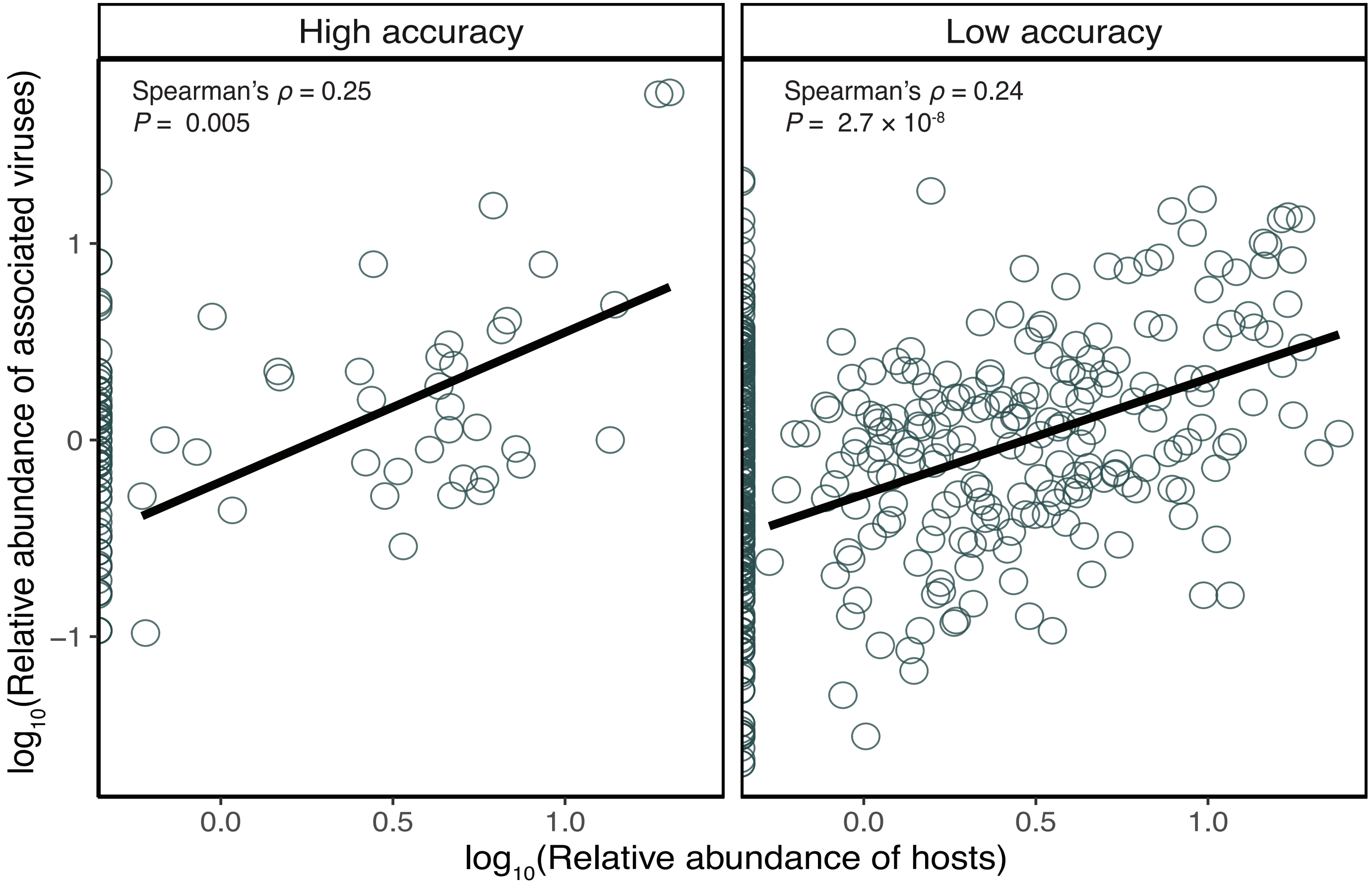

Figure S26. Relationship between the relative abundances of bacterial hosts and their associated viruses in high and low accuracy groups. Correlations were evaluated using Spearman's rank correlation.
